# Atomic-scale insights on hydrogen trapping and exclusion at incoherent interfaces of nanoprecipitates in martensitic steels

**DOI:** 10.1038/s41467-022-31665-x

**Published:** 2022-07-05

**Authors:** Binglu Zhang, Qisi Zhu, Chi Xu, Changtai Li, Yuan Ma, Zhaoxiang Ma, Sinuo Liu, Ruiwen Shao, Yuting Xu, Baolong Jiang, Lei Gao, Xiaolu Pang, Yang He, Guang Chen, Lijie Qiao

**Affiliations:** 1 Beijing Advanced Innovation Center for Materials Genome Engineering, University of Science and Technology Beijing, Beijing, 100083 China; 2 Corrosion and Protection Center, Institute for Advanced Materials and Technology, University of Science and Technology Beijing, Beijing, 100083 China; 3 MIIT Key Laboratory of Advanced Metallic and Intermetallic Materials Technology, Engineering Research Center of Materials Behavior and Design, Ministry of Education, Nanjing University of Science and Technology, Nanjing, 210094 China; 4 School of Computer and Communication Engineering, University of Science and Technology Beijing, Beijing, 100083 China; 5 Department of Materials Science and Engineering, School of Materials Science and Engineering, University of Science and Technology Beijing, Beijing, 100083 China; 6 College of Nuclear Equipment and Nuclear Engineering, Yantai University, Yantai, 264005 China; 7 Beijing Advanced Innovation Center for Intelligent Robots and Systems and Institute of Engineering, Beijing Institute of Technology, Beijing, 100081 China; 8 International School of Advanced Materials, South China University of Technology, Guangzhou, 510640 China

**Keywords:** Structural materials, Techniques and instrumentation

## Abstract

Hydrogen is well known to embrittle high-strength steels and impair their corrosion resistance. One of the most attractive methods to mitigate hydrogen embrittlement employs nanoprecipitates, which are widely used for strengthening, to trap and diffuse hydrogen from enriching at vulnerable locations within the materials. However, the atomic origin of hydrogen-trapping remains elusive, especially in incoherent nanoprecipitates. Here, by combining in-situ scanning Kelvin probe force microscopy and aberration-corrected transmission electron microscopy, we unveil distinct scenarios of hydrogen-precipitate interaction in a high-strength low-alloyed martensitic steel. It is found that not all incoherent interfaces are trapping hydrogen; some may even exclude hydrogen. Atomic-scale structural and chemical features of the very interfaces suggest that carbon/sulfur vacancies on the precipitate surface and tensile strain fields in the nearby matrix likely determine the hydrogen-trapping characteristics of the interface. These findings provide fundamental insights that may lead to a better coupling of precipitation-strengthening strategy with hydrogen-insensitive designs.

## Abstract (web summary)

By trapping hydrogen, nanoprecipitates can mitigate the hydrogen embrittlement of high strength steels. Here, the authors report direct evidences on the structural and chemical features underlying distinct hydrogen-trapping behaviors at the incoherent interfaces of precipitates and steel matrix.

## Introduction

The pledge of zero carbon dioxide emission has provoked an increasing demand on clean hydrogen to reduce the use of fossil fuels. However, hydrogen poses detrimental effects on the mechanical properties of metallic materials^1–6^, commonly known as hydrogen embrittlement (HE), which endangers the safe operation of structural materials such as high-strength alloys in energy-efficient vehicles^7^ and hydrogen storage tanks^8^. Generally, hydrogen embrittlement sensitivity increases with the material strength, being a major concern in the development and practical application of high strength alloys. One of the most attractive methods to intervene and mitigate HE employs nanosized precipitates, which have demonstrated great efficacy in strengthening and toughening materials^9–11^, to trap and diffuse hydrogen atoms from enriching at vulnerable locations in the materials^12–14^.

It is generally believed that hydrogen can be captured inside the alloy carbide nanoparticles and/or in proximity to their interfaces with the matrix, presumably by vacancies, specific interfacial structures, misfit strains, and/or threading dislocations^14–19^. Nevertheless, as for incoherent nanoprecipitates which may provide a deeper trap for hydrogen than the coherent and semi-coherent interfaces^20,21^, it remains contentious as for whether they can trap hydrogen. Thermal desorption spectroscopy (TDS) results indicate that incoherent precipitates such as TiC, NbC, and VC in steels do not trap hydrogen when the hydrogen is electrochemically charged into the material at room temperature^14,20–22^. On the contrary, atom probe tomography (APT) results solidly reveal deuterium segregation at the incoherent interfaces between the NbC and martensite matrix^23^. Depending on the precipitate/matrix mutual orientation, the structures of incoherent interfaces are intrinsically diverse and may presumably lead to different hydrogen-trapping behaviours. Therefore, ensemble-average methods such as the TDS are not sufficient to unravel the hydrogen-trapping characteristics of individual precipitates, not to mention the key underlying mechanisms. On the other hand, though APT can image the spatial distribution of hydrogen, it has to destruct the sample hindering further characterization on the trapping sites^24^.

Here, by using a non-destructive method of scanning Kelvin probe force microscopy (SKPFM) for hydrogen detection, unprecedentedly, we in situ unveil the hydrogen trapping behaviours of individual incoherent nanoprecipitates within a high-strength low-alloyed steel. It is found that not all incoherent interfaces are trapping hydrogen introduced through electrochemical charging; some may even exclude hydrogen. Subsequent characterizations on the precipitates with aberration-corrected scanning transmission electron microscopy (STEM) unravel the structural and chemical origins of the diverse hydrogen trapping behaviours, identifying carbon/sulfur vacancies on the precipitate surface and tensile strain fields in the nearby matrix as key factors that capture hydrogen on the precipitate/matrix interfaces (simply referred to as interface in the following).

## Results

### In situ SKPFM experiment

**Fig. 1:**
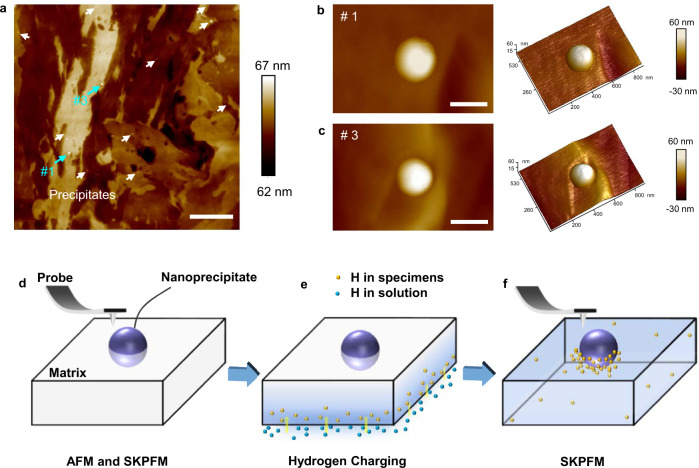
Atom force microscope (AFM) maps of the precipitates in martensite matrix and schematic illustrations of the experiment procedure. **a** Topography map for martensite matrix including nanoprecipitates. **b**, **c**. Tomography maps of precipitate #1 and #3 corresponding to marked by the cyan arrows in panel **a** respectively. **d**–**f**. Schematic illustrations of the scanning Kelvin probe force microscopy (SKPFM) experiment procedure. Scale bars in **a** 4 μm, in **b**, **c** 200 nm.

Figure 1a–c show the atom force microscope (AFM) topography map of the high-strength low-alloyed steel sample, featuring with round-shaped nanoprecipitates uniformly dispersed in the martensite matrix (Supplementary Fig. 1 for a bright-field TEM image of the steel microstructure). After an initial SKPFM scan (Fig. 1d), the plate sample is transferred onto a device for charging hydrogen on the backside for 25 min (Fig. 1e). The electrochemical charging process is carried out in a 0.2 mol L^−1^ NaOH and 0.22 g L^−1^ thiourea electrolyte with a current density of 12 mA cm^−2^. During the process, hydrogen atoms form on the backside of the sample and, driven by the concentration gradient, gradually diffuse along the sample thickness direction to the oxide film on the front surface of the plate sample and then desorb after recombination into hydrogen gas or oxidation in the air into water molecules. The trend of hydrogen content evolution within the sample is roughly simulated (Supplementary Fig. 2 and Supplementary Method 1)^25,26^.

**Fig. 2:**
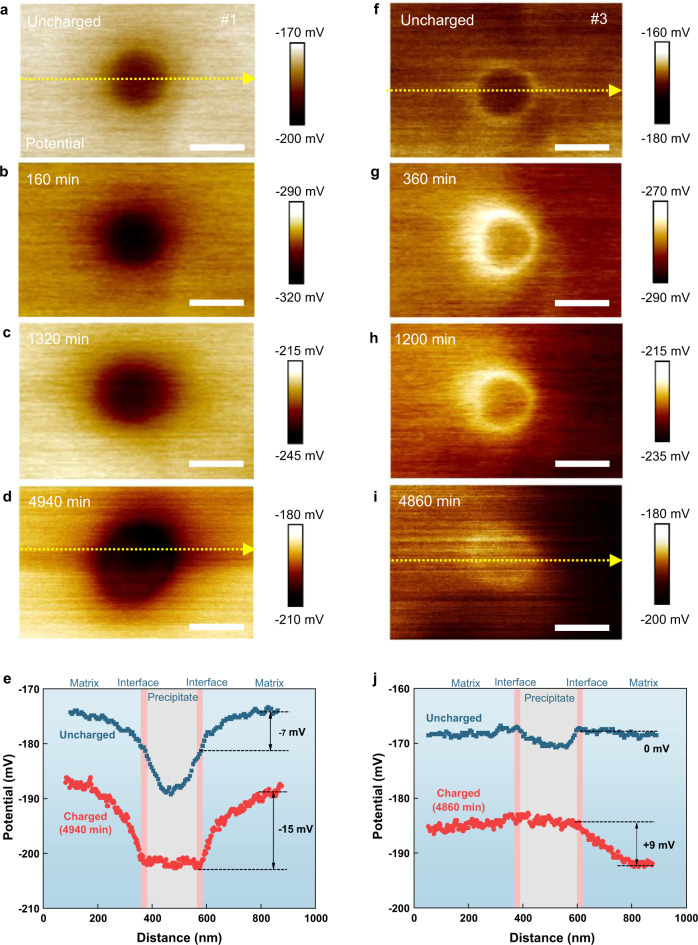
In situ scanning Kelvin probe force microscopy (SKPFM) measurement on hydrogen-precipitate interactions. **a**–**d** Potential maps of precipitate #1 measured at uncharged state and sequential hydrogen-charged states at *t* = 160 min, 1320 min, 4940 min. **e** Potential profiles along the yellow dotted line in panels **a**, **d**. **f**–**i** Potential maps of precipitate #3 measured at uncharged state and sequential hydrogen-charged states at *t* = 360 min, 1200 min, 4860 min. **j** Potential profiles along the yellow dotted line in panels **f**, **i**. All scale bars, 200 nm.

The sample is immediately transferred back for SKPFM imaging after the hydrogen charging (Fig. 1f). Referring to the sample preparation process (see Methods), a thin film of oxides exists on the sample surface which should mainly contains iron oxides, hydroxides, and oxyhydroxides; when hydrogen atoms arrive at the oxide film, they would partially reduce these compounds, leading to a decrease in the work function^27,28^. As such, by detecting the Volta potential difference (simply referred to as “potential” in the following and see Methods for the definition), the in situ SKPFM can actually monitor the gradual infusion and egression of hydrogen as the electrochemically-charged hydrogen ingress on the front surface^29–31^. Thereby, two types of apparently distinct responses at the precipitate/matrix interface are revealed; representative cases of each type are shown in the time-lapsed SKPFM images in Fig. 2 and described in the following. Note that, since hydrogen atoms would likely recombine to desorb or be oxidized by oxygen in air and desorb as water, their activity in the oxide and hence the detected potential drop are not high^31^.

### In situ SKPFM on the hydrogen trapping by the precipitate

Note that before hydrogen charging, both precipitates interiors have lower potential than the martensite matrix (Fig. 2a, f). As hydrogen diffuses toward the front surface, potential of the region gradually decreases and reaches the minimum at ~160 mins (Fig. 2b). Subsequently, when hydrogen desorption overruns ingression, potential of the region gradually increases (Fig. 2c). Incidentally, the simulated hydrogen concentration evolution on the sample surface shows a similar trend (Supplementary Fig. 2), albeit quantitative correlation between hydrogen concentration and the potential is yet to be explored. Remarkably, when hydrogen desorption almost completes at 4940 mins, potential of the interface is the lowest compared with the precipitate interior and the matrix (Fig. 2d). Additionally, compared with the pristine state, the potential drops more steeply at the interface than those at the matrix and the precipitate (Fig. 2e). These phenomena imply that the border area traps more hydrogen than the matrix. Temporal evolution of the measured potential drop also supports this (Supplementary Fig. 3).

### In situ SKPFM on the hydrogen exclusion by the precipitate

In stark contrast to the above observation, the area nearby the border of the other precipitate shows totally different response upon hydrogen ingression (Fig. 2f–j). As hydrogen diffuses toward the front surface, potential of the interface is characteristically higher than those of the matrix and the precipitate interior; this is manifested vividly by the “bright ring” features in the SKPFM images (Fig. 2g, h). This feature remains even when hydrogen desorption almost completes at 4860 min (Fig. 2i), suggesting that the area nearby the border of this precipitate is less prone to trap hydrogen than the matrix.

Note that the above distinct scenarios of precipitate-hydrogen interaction are repeatedly found in several independent tests with different nanoprecipitates (Supplementary Fig. 4). Particularly, 240 hrs after the initial hydrogen charging, the potential of the interface of precipitate #2 is still lower than that of the matrix (Supplementary Fig. 4c), suggesting that the interface provides a deep trap for hydrogen. Since precipitates with different hydrogen trapping behaviours have been found simultaneously in the same martensite lath (Supplementary Fig. 4e, f), these distinct behaviours should not be attributed to the matrix. To pinpoint the underlying factor that governs the hydrogen-trapping characteristics of these precipitates, in the following, we compare the phases of the precipitates and atomic-scale structural and chemical features of the interface.

### Precipitate phase analysis

**Fig. 3:**
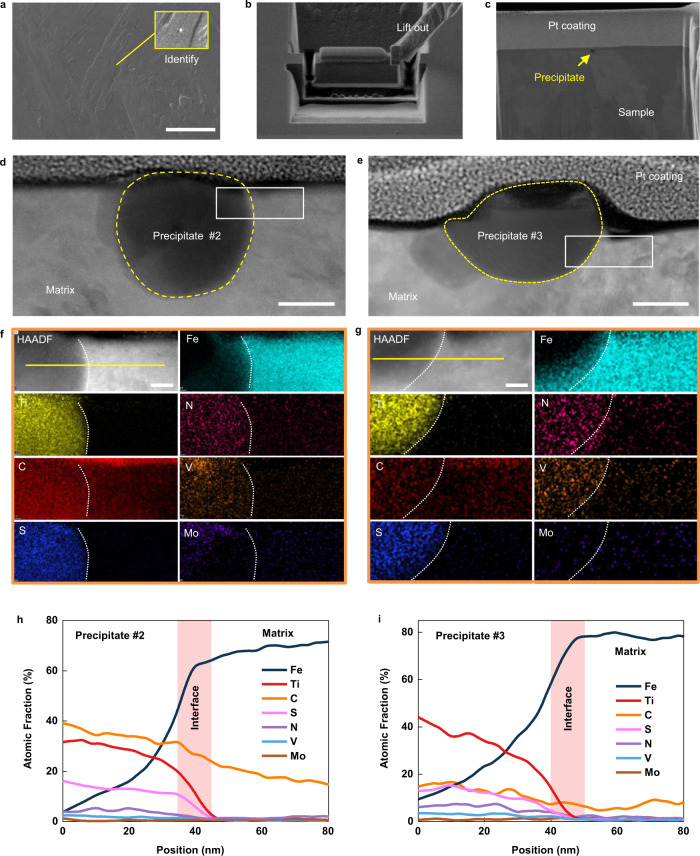
Lift-out and scanning transmission electron microscopy energy dispersed X-ray spectroscopy (STEM-EDS) analysis of the precipitates. **a–c** Identify, lift out, and FIB thinning of the precipitates. **d**, **e** STEM images of the precipitates #2 and #3, respectively. **f**, **g** EDS mapping of the boxed regions in panels **d**, **e**. **h**, **i** EDS line data along the yellow line in panels **f**, **g** showing the elemental composition across the precipitate/matrix interface. Note that the EDS quantification on C content is not accurate due to the carbon contamination that is commonly encountered in STEM imaging. Scale bars in **a** 10 μm, in **d**, **e** 50 nm, in **f**, **g** 20 nm.

**Fig. 4:**
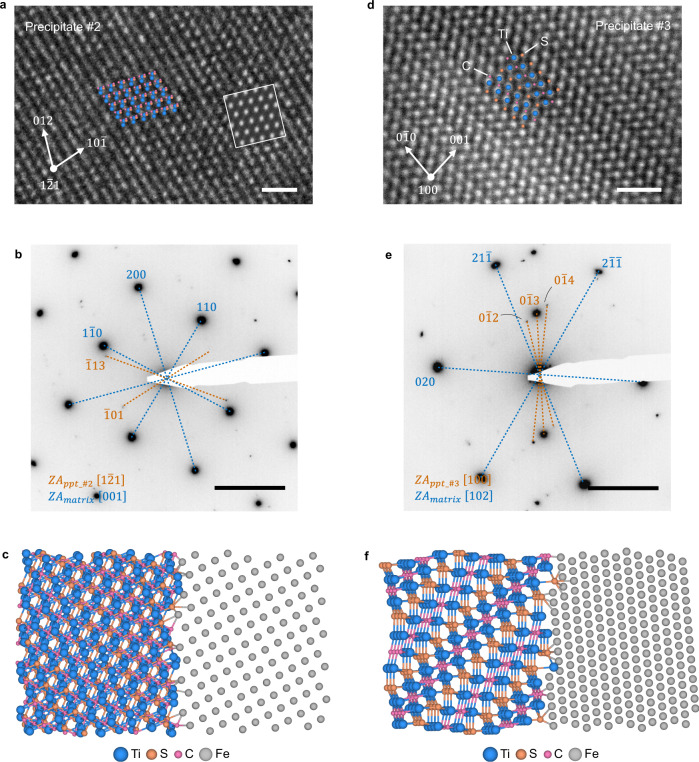
Structure of the precipitate and the precipitate/matrix interface. **a–c** High-resolution transmission electron microscopy (HRTEM) image of the precipitate #2, electron diffraction pattern of its interface with the matrix, and atomic structure model of the interface constructed based on the mutual orientation, respectively. **d**–**f** Scanning transmission electron microscopy (STEM) image of the precipitate #3, electron diffraction pattern of its interface with the matrix, and atomic structure model of the interface constructed based on the mutual orientation, respectively. Note that, in panel **e**, the precipitate was not oriented exactly on the [100] zone axis but tilted 7.6° away from the zone axis (Supplementary Fig. 5); as such, the diffraction spot of the (010) plane is invisible; the diffraction spot of the (001) in panel **e** is a forbidden reflection visible due to double diffraction. Insets in panels **a, d** are corresponding projection view of the Ti_2_CS atomic models and simulated HRTEM images. Scale bar in **a**, **d**, 1 nm, in **b**, **e** 5 nm^−1^.

By using the focused ion beam (FIB), the precipitates and surrounding matrix are lifted out and thinned for STEM characterization (Fig. 3a–c). Note that precipitate #2 with the same hydrogen-trapping characteristic as precipitate #1 is chosen for the analysis (see its SKPFM result in Supplementary Fig. 4c and Supplementary Note 1). First of all, as shown by the high-angle annular dark-field scanning transmission electron microscopy (HAADF-STEM) images and energy-dispersive X-ray spectroscopy (EDS), the two precipitates have similar composition of mainly Ti, C, and S (Fig. 3d–i). Note that the EDS quantification on C content is not accurate due to the carbon contamination that is commonly encountered in STEM imaging. Further, high-resolution transmission electron microscopy (HRTEM), STEM and the selected area electron diffractions (SAED) of the precipitates are acquired (Fig. 4a, b, d, e). After scrutinizing all reported phases containing Ti-C-S, Ti-C, or Ti-S, it is found that only the Ti_2_CS phase (space group P63/mmc, lattice parameters of *a* = 0.32 nm and *c* = 1.13 nm) provides the best match to all the experimental data including the HRTEM image, STEM image, SAED patterns, and EDS quantification results on Ti/S ratio of the precipitates.

### Structures of the precipitate/matrix interfaces

Mutual orientations of the precipitate and the surrounding matrix are determined by using the SAED analysis. The [$1\bar{2}1$]_Ti2CS_ zone axis of precipitate #2 is roughly parallel to the [001]_matrix_ axis of the matrix (Fig. 4b); the [100]_Ti2CS_ axis of precipitate #3 tilts 7.6° away from the [102]_matrix_ axis of the matrix (Fig. 4e and Supplementary Fig. 5 for more details on determining the mutual orientation). These orientation relation between the precipitate and the martensite matrix is neither the Baker–Nutting orientation relationship^32^ nor the Nishiyama-Wassermann (N-W) orientation relationship^33^ that are conventionally found in coherent or semi-coherent TiC precipitates. Based on the mutual orientation analysis, atomic models of the interfaces are manually constructed by using an QuantumATK software^34^, which clearly illustrated the incoherent precipitates/matrix interfaces (Fig. 4c, f). Note that these models are only for illustration purposes and requires atomic-scale characterization in 3 dimensions to be accurate^35,36^.

### Chemical features of the precipitate/matrix interfaces

**Fig. 5:**
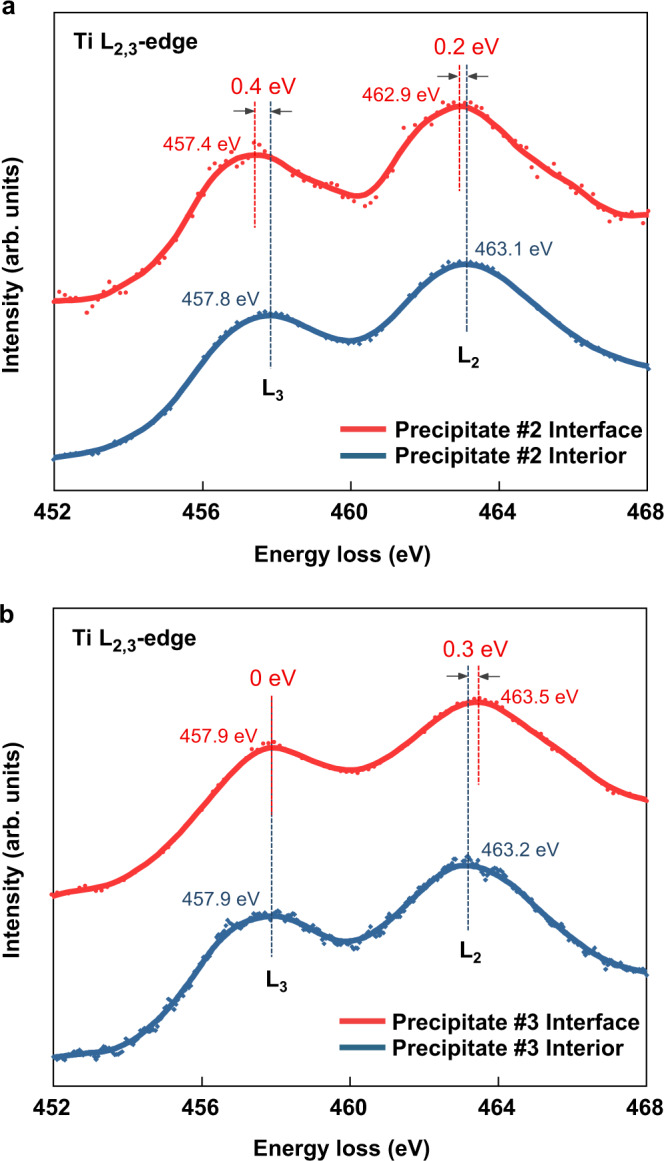
Electron energy loss spectroscopy (EELS) analysis on the Ti L_2, 3_ core-loss edges of precipitate interior and interface with the martensite matrix. **a** EELS spectrums of precipitate #2 interior and interface. **b** EELS spectrums of precipitate #3 interior and interface. Dash lines indicate the L_2_ and L_3_ peak positions.

Note that the EDS line-scan data across the interface indicates no significant elemental segregation at the interfaces (Fig. 3h, i). To explore possible carbon or sulfur vacancies on the interface, we performed electron energy loss spectroscopy (EELS) analysis on the L_2, 3_ core-loss edges of Ti for precipitates #2 (Fig. 5a) and #3 (Fig. 5b). Comparing the precipitation #2 interior Ti-L_2, 3_ peaks (463.1 eV for Ti-L_2_ and 457.8 eV for Ti-L_3_), the interface Ti-L_2, 3_ peaks shift 0.2 eV and 0.4 eV, respectively to lower energies. As for the precipitate #3, the interface Ti-L_2, 3_ peaks shift 0.3 eV and 0 eV respectively to higher energies, compared with the precipitation interior (463.2 eV for Ti-L_2_ and 457.9 eV for Ti-L_3_). Referring to literatures^37^, shifting of the Ti-L_2, 3_ peaks to lower energies corresponds to a decrease of the Ti valence state. Therefore, the above EELS data indicates that Ti on the interface of precipitation #2 and matrix is more reduced than Ti on the interface of precipitation #3 and matrix, likely due to the presence of more C or S vacancies.

To evaluate the effect of C and S vacancies on the hydrogen trapping ability, we performed density function theory (DFT) calculations on the solution energy of H atom at C and S vacancies within the Ti_2_CS precipitate (see Methods for details). As shown in Table 1, the calculated solution energy of hydrogen at C vacancy and S vacancy are −0.75 eV and −0.38 eV, respectively; while that of α-Fe (tetrahedral site) is markedly higher (0.23 eV). An interesting question arises as how the area nearby the border of precipitate #3 excludes hydrogen even though it may not contain as much C/S vacancies as the interface of precipitate #2 and the matrix.

**Table 1:** The density function theory (DFT) and nudged elastic band (NEB) calculation results.

Hydrogen trapping sites	H solution energy [eV]	Tetrahedral volume [Å^3^]	H diffusion barrier [eV]
α-Fe	0.23	2.18	0.101
Strain state of precipitate #2	0.05	2.24	0.097
Strain state of precipitate #3	0.32	2.14	0.113
C vacancy	−0.75	–	–
S vacancy	−0.38	–	–

### Strain state on the precipitate/matrix interfaces

**Fig. 6:**
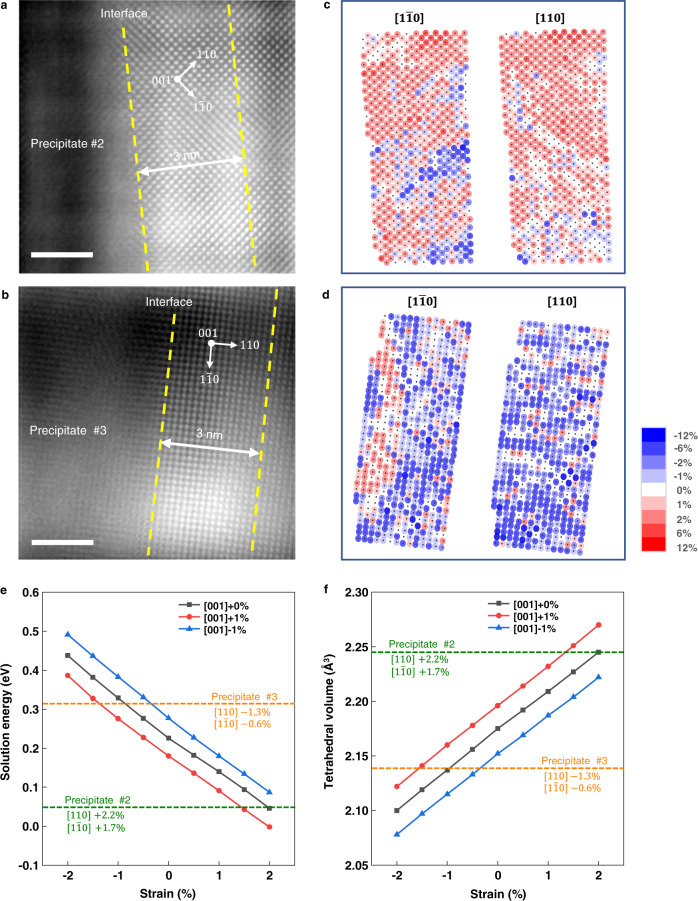
Atomic strain maps nearby the border of the precipitates. **a**, **b** The filtered atomic resolution high-angle annular dark field scanning transmission electron microscopy (HAADF-STEM) images of the martensite matrix nearby the interfaces. Yellow dash lines mark the region for the atomic strain analysis. Note that, in each case, the reference region is within the same martensite lath and ~150 nm afar from the interface (Supplementary Fig. 6). **c**, **d** Atomic strain maps of the region within 3 nm distance from the interfaces. **e**, **f** Density function theory (DFT) calculations on the solution energy of H atom at the tetrahedral interstitial sites and corresponding tetrahedral volume in α-Fe of precipitate #2 (green dash lines) and precipitate #3 (yellow dash lines). Note that the same magnitude of strains are applied to the [110] and $[1\bar{1}0]$ directions; and the positive and negative values of strain represent tensile and compressive strains, respectively. The [001]+1% notion in the legend means that an additional 1% tensile strain was applied along the [001] direction. Scale bars in panels **a**, **b**, 2 nm.

The strain state of the matrix right next to the interface is analyzed by directly interpreting the atoms-column positions in atomic-resolution HAADF-STEM images. We used the CalAtom^38^ software to determine the precise position of each atomic column within the region of without obvious dislocations to avoid the influence of dislocation cores on the strain measurement (Fig. 6a, b and Supplementary Fig. 6). Note that the CalAtom software employs a multiple-ellipse fitting algorithm to determine the positions of the atomic columns with high precision, which has been widely applied in literatures^39,40^. Following the strain analysis method in the literature^41^, atomic strain maps are generated by comparing the nearest-neighbor distances in two crystallographic directions (namely $\left[1\bar{1}0\right]$ and [110]) separately to their reference values. For visualization, the strain at each atomic column is color-coded and plotted into a 2D map (Fig. 6c, d). We measured and compared the strain values of the martensite matrix within 3 nm from the interface (Fig. 6a, b) and 150 nm afar from the interface (Supplementary Fig. 6a, b). Separate strain maps are generated for each of $\left[1\bar{1}0\right]$ and [110] crystallographic directions. All strain values within the area of interest are statistically plotted (Supplementary Fig. 7a). The statistical measurements suggest that, the lattices nearby the border of precipitate #2 is tensile-strained by, on average, 2.2% along the [110] direction and 1.7% along the $\left[1\bar{1}0\right]$ direction; as for the precipitate #3, the region right by the border is compressively strained by, on average, −1.3% along the [110] direction and −0.6% along the $\left[1\bar{1}0\right]$ direction.

To further evaluate the effects of above strains on the hydrogen trapping, we carried out DFT calculations on the solution energy of H atom at the tetrahedral interstitial site and the tetrahedral volume of α-Fe at the above strained states (see Methods for details). As shown in Table 1, the solution energy of tensile strained α-Fe is less than that of compressively strained α-Fe, while the tetrahedral volume of the former is larger than the latter, implying that the strain state corresponding to the case of precipitate #2 is more favorable to trap hydrogen than that of the precipitate #3. In other words, the tensile strain fields facilitate the dissolution of H while the compressive strain suppress it. When additional 1% tensile/compressive strain was applied along the [001] direction (i.e., parallel to the observation direction of the TEM experiment) in the DFT calculation, the above finding persists (Fig. 6e, f).

## Discussion

It is well known that the solution of hydrogen will lower the work function of the oxide film on the sample surface^27,28^ and hence lowering the potential as detected by the SKPFM^42–44^. Note that the surface species on the Ti_2_CS precipitates and the martensite matrix are likely different, and hence their responses to hydrogen may not be the same; therefore, we cannot conclude on the relative H concentration within the precipitate by comparing the potential changes with those on the matrix. However, the changes in potential of precipitate interior decreasing and recovering in the same way by the infusion and egression of hydrogen need consideration. H flux may have entered the Ti_2_CS precipitates and contributed to the measured potential drop at the precipitate surface during the in situ SKPFM experiments. In principle, H atoms entering the precipitates will not affect the trapping and diffusion of H on the interfaces. (Supplementary Discussion 1 for more on this point).

Nevertheless, the oxide film in the vicinity of the interface is reasonably the same as that on the matrix afar (Supplementary Discussion 2 and Supplementary Fig. 8); and hence their responses to H should be the same. The SKPFM results of precipitate #1 showing the lowest potential and largest potential drop at the interface (Fig. 2a–e and Supplementary Fig. 3a, b) indicate that hydrogen prefers to lodge in the vicinity of the incoherent interface than within the martensite matrix. Whereas the SKPFM results of precipitate #3, showing higher potential at the interface than within the martensite matrix (Fig. 2f–j and Supplementary Fig. 3c, d), suggest that the interface tend to exclude hydrogen compared with the martensite matrix. More discussions on how we interpreted the SKPFM results can be found in Supplementary Discussion 3. As corroborated by the repeated results on other precipitates (Supplementary Fig. 4), the incoherent interfaces are apparently not reacting in the same way upon the infusion of hydrogen. This finding can satisfactorily explain the seemingly controversial discoveries in macro- and micro-scale experiments. As for bulk materials wherein a large number of incoherent precipitates coexist, the ensemble-averaged hydrogen trapping ability of the precipitates could be trivial as reflected in many TDS results^20,21^. However, when probing individual precipitates, it is fairly possible to capture hydrogen-trapping by incoherent interfaces^23^.

Hydrogen-trapping ability of precipitates are conventionally attributed to atomic-scale features of the interface including chemical features such as carbon vacancies^45^ and structural features such as misfit dislocations^5,14–16^ and strains^46^. Though reasonably demonstrated in (semi)coherent precipitates, this is implied more often than proven in incoherent precipitates. The diverse hydrogen trapping behaviours may root in the fact that the incoherent interfaces easily vary with precipitate-matrix mutual orientations and the precipitate formation conditions^15,47^. Therefore, pin-pointing the mechanism of hydrogen trapping by the incoherent interfaces indispensably requires characterizations of the atomic-scale structure and hydrogen trapping behaviours with the same single precipitate, as uniquely enabled here through the in situ SKPFM-FIB-TEM workflow.

On chemical features of the interface, the quantitative EDS line-scan results in Fig. 3h, i indicate no obvious elemental segregation on the both interfaces. However, the EELS data shows a minor shift in the Ti L_2, 3_ peaks positions, indicating that the valence state of Ti on the surface of precipitate #2 is lower than that of precipitate #3^37^. This implies that the C and/or S vacancies exist on the surface of precipitate #2. In fact, carbon vacancies are commonly found in alloy carbide precipitates in ferritic steels as have been demonstrated in previous APT results showing the atomic ratios of C and transition metal of the precipitates being lower than the ideal stoichiometry^45^; in addition, the formation energy of carbon vacancies decreases with the loss of coherency at the TiC/α-Fe interface^46^. The underlying reason for the different contents of surface C/S vacancies may be associated with the formation temperature of the precipitates. Thermal dynamic calculations have proven that concentration of carbon vacancies in alloy carbide precipitates increases with temperature^48^. Given that the incoherent Ti_2_CS precipitates can be formed in a wide temperature window (Supplementary Fig. 9), the C/S vacancies are deemed to present with varying concentrations in different precipitates. Additionally, concentration fluctuation of Ti, C, and/or S at the precipitate formation site can also incur the non-stoichiometry at the interface.

Due to the existing difficulty in reasonably constructing the intrinsically non-periodical structure of the incoherent interface, we cannot calculate the solution energies of C/S vacancies on the precipitate surface. Though, our previous studies on coherent interfaces between α-Fe and other precipitates (VC, TiN, NbC) suggest that the solution energies of non-metallic element vacancies on the interface are considerably lower than those inside the bulk precipitate^17^. Moreover, extensive theoretical works suggest that carbon vacancies on the surface of alloy carbide precipitates are deep hydrogen traps^17,45,49^, and reducing the amount of surface carbon vacancies may lower their hydrogen trapping energy^45^. As such, the C and/or S vacancies on the surface of precipitate #2 are deemed to impart a better hydrogen trapping capability than the surface of precipitate #3. Moreover, the C and S vacancies on the precipitate surface are readily accessible to hydrogen^45,46^.

On the atomic structure feature of the interface, firstly, mutual orientation analysis shows that interfaces of both precipitates are incoherent in nature (Fig. 4). Due to the lack of effective experimental or computational approaches, it is hard to solidly unravel the atomic structures of the interfaces. Nevertheless, as illustrated in Fig. 4c, f, the two interfaces should have different structures with different interstitial sites for anchoring hydrogen. Secondly, strain analysis indicates that the martensite matrix nearby the precipitate #2 are tensile strained while that in the vicinity of precipitate #3 are compressively strained (Fig. 6). As corroborated by the DFT calculations, the tensile strain field prompts hydrogen trapping while the compressive strain field nearby precipitate #3 suppresses hydrogen trapping, which are consistent with the SKPFM findings and indicates that tensile strain field is a hydrogen trapping mechanism at the vicinity of incoherent interfaces. Though, the DFT calculations and previous reports^46^ suggest that the strain fields are weak hydrogen traps than the C/S vacancies (Table 1). The different strain fields surrounding different precipitates may be associated with the atomic structures between the Ti_2_CS precipitates and the martensite matrix. Moreover, the hydrogen-excluding behaviours as shown on the border of precipitate #3 may be associated with the fact that the compressive strains in the nearby matrix increase the solution energy of H.

Due to the limitation of the SKPFM resolution, we only probed precipitates of 100 nm or larger in diameter, which are usually formed in the smelting and hot-rolling process in the high temperature range. Though, the findings on the atomistic origins of hydrogen-trapping/exclusion at the precipitates interface are directly applicable to much smaller precipitates with semi-coherent or coherent interfaces wherein non-stoichiometry and elastic strains are common^20,45^, thusly paving way for achieving desirable precipitate-hydrogen interaction in steels and coupling of precipitation-strengthening and hydrogen-insensitive designs. For instance, to trap hydrogen from concentrating at vulnerable locations and hence mitigate hydrogen embrittlement, precipitates in steels could be designed with non-stoichiometry and/or desirable strain fields in the surrounding matrix. In fact, this may have been realized in literatures reporting the positive roles of elastic-strain field^17,20^, interfacial dislocations^47^, and vacancies^45^ around the coherent or semi-coherent precipitates in lowering the hydrogen embrittlement sensitivity of steels. With the advancement of SKPFM resolution^44^, the experimental methods as demonstrated here may enable direct investigation on much smaller precipitates.

Above all, the dynamic interactions between hydrogen and incoherent precipitates are captured. Atomic-scale structural and chemical features of the very interfaces suggest that carbon/sulfur vacancies on the precipitate surface and strain of the matrix nearby the interface determine the hydrogen-trapping characteristics of the incoherent interfaces. The findings unravel the perplexing hydrogen trapping behaviours of incoherent precipitates. Since direct imaging of solute hydrogen atoms is difficult^50^, our study demonstrates a viable method to directly probe the hydrogen trapping behavior and the structural/chemical characteristics of exactly the same site, paving way for the study on the atomic origin of hydrogen-assisted cracking, especially when structures of the associated defects (such as grain boundaries and matrix/inclusion interfaces) are intrinsically diverse.

## Methods

### Materials

A high-strength low-alloy martensitic steel was used for this study. Composition of the steel is shown in Supplementary Table 1. The steel was hot-rolled to 12.7 mm and then cold-rolled to 10 mm. The plate was solution treated at 900 °C for 30 min, quenched in water, tempered at 500 °C for 60 min and then cooled in air. A bright-field TEM image of the martensitic steel is shown in Supplementary Fig. 1. The hydrogen diffusion coefficient of the steel is measured to be 3.3 × 10^−8^ cm^2^ s^−1^.

### In-situ SKPFM experiment procedure

The steel was cut into plates of 0.49 mm in thickness; the plate specimen was mechanically polished with SiO_2_ suspension and then etched with Argon ion for 30 mins to remove the native oxide and contaminations on the surface. Thereafter, when the fresh surface was exposed to air, it should have been oxidized again by the oxygen in air, forming a very thin oxide film on the sample surface. The SKPFM experiments were conducted in the tapping mode using a dimension Nanoscope V (Veeco Instruments Inc.). The Volta potential difference (simply referred to as potential in the main text) was obtained in situ before and after hydrogen charging, and is defined by (*φ*_sample_ – *φ*_tip_)/*e*, where *φ*_sample_ and *φ*_tip_ are the work function of sample and tip, respectively, and *e* represents the value of the elementary charge^43,51^. In SKPFM, *φ*_tip_ is a constant; therefore, the potential is proportional to *φ*_sample_ which changes with the local hydrogen concentration^31^. The cantilever used in our study has a spring constant of 2.8 N m^−1^, a resonant frequency of 60–100 kHz, and a standard PtIr-coated silicon tip with a <25 nm curvature. Lift height was set to 60 nm. The SKPFM experiments were carried out in air at room temperature and relative humidity of 38 ± 1%. The system was calibrated by using a high-quality highly-oriented pyrolytic graphite (HOPG)^52^ prior to the experiment to ensure the accuracy of the potential measurements.

### TEM sample preparation and characterization

The TEM sample was prepared by using a Thermo Fisher Helios dual-beam system. To protect the precipitate from damage, carbon, and platinum layers were deposited with the electron beam on top of the precipitate before the FIB milling. TEM, STEM, and EDS analysis were performed on a Thermo Fisher ThemisZ TEM equipped with both probe and imaging lens spherical aberration correctors. EELS was acquired with a Gatan GIF Continuum 1065 instrument.

### Density Function Theory calculations

The DFT calculations were performed in the Vienna ab initio Simulation Package (VASP)^53^. Hydrogen diffusion energy barrier were mapped by the minimum energy reaction path with the nudged elastic band (NEB)^54^ method. The core electrons were modeled with the projector-augmented-wave (PAW) method^55^. The exchange and correlation functional of Perdew-Burke-Ernzerhof (PBE) was adopted within the framework of generalized gradient approximation (GGA)^56^. An energy cut-off of 400 eV and 5 × 5 × 5, 3 × 3 × 3 k-point meshes were used to ensure calculation accuracy for α-Fe and Ti_2_CS, respectively. All of the calculations were carried out as spin-polarized and the forces on all the atoms were less than 0.02 eV Å^−1^ during the geometrical optimizations. During the transition state calculation, the atoms were allowed to fully relaxed until the forces on all atoms were less than 0.05 eV Å^−1^. The total energy was converged to 10^−5^ eV for the Self-Consistent Field (SCF) calculation.

The solution energy of an H atom at the trapping sites (tetrahedral interstitial site of α-Fe, C vacancy and S vacancy in Ti_2_CS precipitate) is defined by,

1 $${E}_{{{{{{\rm{sol}}}}}}}={E}_{{{{{{\rm{trap}}}}}}+{{{{{\rm{H}}}}}}}-{E}_{{{{{{\rm{trap}}}}}}}-\frac{1}{2}{E}_{{{{{{{\rm{H}}}}}}}_{2}}$$

where *E*_trap+H_ is the total energy of the unit-cell that dissolves one H atom, *E*_trap_ is the total energy of the same unit-cell without H, and ${E}_{{{{{{{\rm{H}}}}}}}_{2}}$ is the energy of an isolated hydrogen molecule. The atomic structures used in the above calculation were shown in Supplementary Fig. 10.

## Supplementary information

Supplementary Information

## Data Availability

All the data related to this manuscript have been included in the main text and supplementary information. All the raw data are stored in University of Science and Technology Beijing and is available upon request from the correponding authors Y.H. and L.Q.

## References

[1] Hirth JP (1980). Effects of hydrogen on the properties of iron and steel. Metall. Trans. A.

[2] Qiao LJ, Luo JL, Mao X (1998). Hydrogen evolution and enrichment around stress corrosion crack tips of pipeline steels in dilute bicarbonate solution. Corrosion.

[3] Song J, Curtin WA (2013). Atomic mechanism and prediction of hydrogen embrittlement in iron. Nat. Mater..

[4] Raabe D, Tasan CC, Olivetti EA (2019). Strategies for improving the sustainability of structural metals. Nature.

[5] Shi R (2020). Atomic-scale investigation of deep hydrogen trapping in NbC/α-Fe semi-coherent interfaces. Acta Mater..

[6] Nagao A, Smith CD, Dadfarnia M, Sofronis P, Robertson IM (2012). The role of hydrogen in hydrogen embrittlement fracture of lath martensitic steel. Acta Mater..

[7] Sun B (2021). Chemical heterogeneity enhances hydrogen resistance in high-strength steels. Nat. Mater..

[8] Schlapbach L, Züttel A (2001). Hydrogen-storage materials for mobile applications. Nature.

[9] Yang Y (2021). Bifunctional nanoprecipitates strengthen and ductilize a medium-entropy alloy. Nature.

[10] Tang S (2019). Precipitation strengthening in an ultralight magnesium alloy. Nat. Commun..

[11] Jiang S (2017). Ultrastrong steel via minimal lattice misfit and high-density nanoprecipitation. Nature.

[12] Chen YS (2017). Direct observation of individual hydrogen atoms at trapping sites in a ferritic steel. Science.

[13] Nagao A, Martin ML, Dadfarnia M, Sofronis P, Robertson IM (2014). The effect of nanosized (Ti,Mo)C precipitates on hydrogen embrittlement of tempered lath martensitic steel. Acta Mater..

[14] Wei, F. G., Hara, T. & Tsuzaki, K. Nano-Preciptates Design with Hydrogen Trapping Character in High Strength Steel. (Springer, Berlin Heidelberg, 2011).

[15] Wei FG, Hara T, Tsuzaki K (2004). High-resolution transmission electron microscopy study of crystallography and morphology of TiC precipitates in tempered steel. Philos. Mag..

[16] Takahashi J, Kawakami K, Kobayashi Y, Tarui T (2010). The first direct observation of hydrogen trapping sites in TiC precipitation-hardening steel through atom probe tomography. Scr. Mater..

[17] Ma Y (2020). A first-principles study on the hydrogen trap characteristics of coherent nano-precipitates in α-Fe. Int. J. Hydrog. Energy.

[18] Ohnuma M, Suzuki J, Wei FG, Tsuzaki K (2008). Direct observation of hydrogen trapped by NbC in steel using small-angle neutron scattering. Scr. Mater..

[19] Takahashi J, Kawakami K, Tarui T (2012). Direct observation of hydrogen-trapping sites in vanadium carbide precipitation steel by atom probe tomography. Scr. Mater..

[20] Wei FG, Tsuzaki K (2006). Quantitative analysis on hydrogen trapping of TiC particles in steel. Metall. Mater. Trans. A.

[21] Wei FG, Tsuzaki K (2004). Hydrogen absorption of incoherent TiC particles in iron from environment at high temperatures. Metall. Mater. Trans. A.

[22] Wallaert E, Depover T, Arafin M, Verbeken K (2014). Thermal desorption spectroscopy evaluation of the hydrogen-trapping capacity of NbC and NbN Precipitates. Metall. Mater. Trans. A.

[23] Chen YS (2020). Observation of hydrogen trapping at dislocations, grain boundaries, and precipitates. Science.

[24] Koyama M (2017). Recent progress in microstructural hydrogen mapping in steels: quantification, kinetic analysis, and multi-scale characterisation. Mater. Sci. Technol..

[25] Ma ZX (2018). Experimental study on the diffusion of hydrogen along individual grain boundaries in nickel. Electrochem. commun..

[26] Ma Z, Xiong X, Chen L, Su Y (2021). Quantitative calibration of the relationship between Volta potential measured by scanning Kelvin probe force microscope (SKPFM) and hydrogen concentration. Electrochim. Acta.

[27] Krasemann M, Streckel H, Hoffmann K, Grabke H-J, Stratmann M (1998). Detection of hydrogen ingress into iron oxide and iron oxy-hydroxide layers by the Kelvin probe. Proc. Electrochem. Soc..

[28] Evers S, Senoz C, Rohwerder M (2013). Hydrogen detection in metals: a review and introduction of a Kelvin probe approach. Sci. Technol. Adv. Mater..

[29] Evers S, Senöz C, Rohwerder M (2013). Spatially resolved high sensitive measurement of hydrogen permeation by scanning Kelvin probe microscopy. Electrochim. Acta.

[30] Hua Z (2019). The finding of hydrogen trapping at phase boundary in austenitic stainless steel by scanning Kelvin probe force microscopy. Scr. Mater..

[31] Senöz C, Evers S, Stratmann M, Rohwerder M (2011). Scanning Kelvin Probe as a highly sensitive tool for detecting hydrogen permeation with high local resolution. Electrochem. commun..

[32] Baker RG, Brandon DG, Nutting J (1959). The growth of precipitates. Philos. Mag..

[33] Dahmen U (1981). Orientation relationships in precipitation systems. Acta metall..

[34] Smidstrup S (2019). QuantumATK: an integrated platform of electronic and atomic-scale modelling tools. J. Phys. Condens. Matter.

[35] Zhou J, Yang Y, Ercius P, Miao J (2020). Atomic electron tomography in three and four dimensions. MRS Bull..

[36] Chen CC (2013). Three-dimensional imaging of dislocations in a nanoparticle at atomic resolution. Nature.

[37] Cao N (2019). Doping strain induced bi-Ti3+ pairs for efficient N_2_ activation and electrocatalytic fixation. Nat. Commun..

[38] Zhang Q, Zhang LY, Jin CH, Wang YM, Lin F (2019). CalAtom: A software for quantitatively analysing atomic columns in a transmission electron microscope image. Ultramicroscopy.

[39] Du K (2019). Manipulating topological transformations of polar structures through real-time observation of the dynamic polarization evolution. Nat. Commun..

[40] Niu K (2021). Direct visualization of large-scale intrinsic atomic lattice structure and its collective anisotropy in air-sensitive monolayer 1T’- WTe2. Adv. Sci..

[41] Nilsson Pingel T, Jorgensen M, Yankovich AB, Gronbeck H, Olsson E (2018). Influence of atomic site-specific strain on catalytic activity of supported nanoparticles. Nat. Commun..

[42] Li M, Guo LQ, Qiao LJ, Bai Y (2012). The mechanism of hydrogen-induced pitting corrosion in duplex stainless steel studied by SKPFM. Corros. Sci..

[43] Rohwerder M, Turcu F (2007). High-resolution Kelvin probe microscopy in corrosion science: Scanning Kelvin probe force microscopy (SKPFM) versus classical scanning Kelvin probe (SKP). Electrochim. Acta.

[44] Evers S, Rohwerder M (2012). The hydrogen electrode in the “dry”: A Kelvin probe approach to measuring hydrogen in metals. Electrochem. commun..

[45] Takahashi J, Kawakami K, Kobayashi Y (2018). Origin of hydrogen trapping site in vanadium carbide precipitation strengthening steel. Acta Mater..

[46] Kawakami K, Matsumiya T (2012). Numerical analysis of hydrogen trap state by TiC and V4C3 in bcc-Fe. ISIJ Int..

[47] Shi R (2021). Quantitative investigation on deep hydrogen trapping in tempered martensitic steel. J. Alloy. Compd..

[48] Wei FG, Tsuzaki K (2012). Hydrogen Trapping Phenomena in Martensitic Steels..

[49] Di Stefano D (2016). First-principles investigation of hydrogen interaction with TiC precipitates in α-Fe. Phys. Rev. B.

[50] Graaf SD, Momand J, Mitterbauer C, Lazar S, Kooi BJ (2020). Resolving hydrogen atoms at metal-metal hydride interfaces. Sci. Adv..

[51] Melitz W, Shen J, Kummel AC, Lee S (2011). Kelvin probe force microscopy and its application. Surf. Sci. Rep..

[52] Örnek C, Engelberg DL (2015). SKPFM measured Volta potential correlated with strain localisation in microstructure to understand corrosion susceptibility of cold-rolled grade 2205 duplex stainless steel. Corros. Sci..

[53] Kresse G, Furthmuller J (1996). Efficient iterative schemes for ab initio total-energy calculations using a plane-wave basis set. Phys. Rev. B.

[54] Henkelman G, Uberuaga BP, Jónsson H (2000). A climbing image nudged elastic band method for finding saddle points and minimum energy paths. J. Chem. Phys..

[55] Blöchl PE (1994). Projector augmented-wave method. Phys. Rev. B.

[56] Perdew JP, Burke K, Ernzerhof M (1996). Generalized gradient approximation made simple. Phys. Rev. Lett..

