# Supplementary material for: Atomic-scale insights on hydrogen trapping and exclusion at incoherent interfaces of nanoprecipitates in martensitic steels
Source: Nat Commun. 2022 Jul 5;13:3858. doi: 10.1038/s41467-022-31665-x (PMC9256589; doi:10.1038/s41467-022-31665-x)
Supplement: Supplementary file 1 — Supplementary Information [file 41467_2022_31665_MOESM1_ESM.pdf]

Supplementary Information for

Atomic-scale insights on hydrogen trapping and exclusion at incoherent interfaces of nanoprecipitates in martensitic steels

B. Zhang *et al.*

<u>Inventory of Supporting Information</u>

Supplementary Figs. 1-10

Supplementary Table 1

Supplementary Method 1

Supplementary Note 1

Supplementary Discussions 1-3

Supplementary References

Supplementary Figures

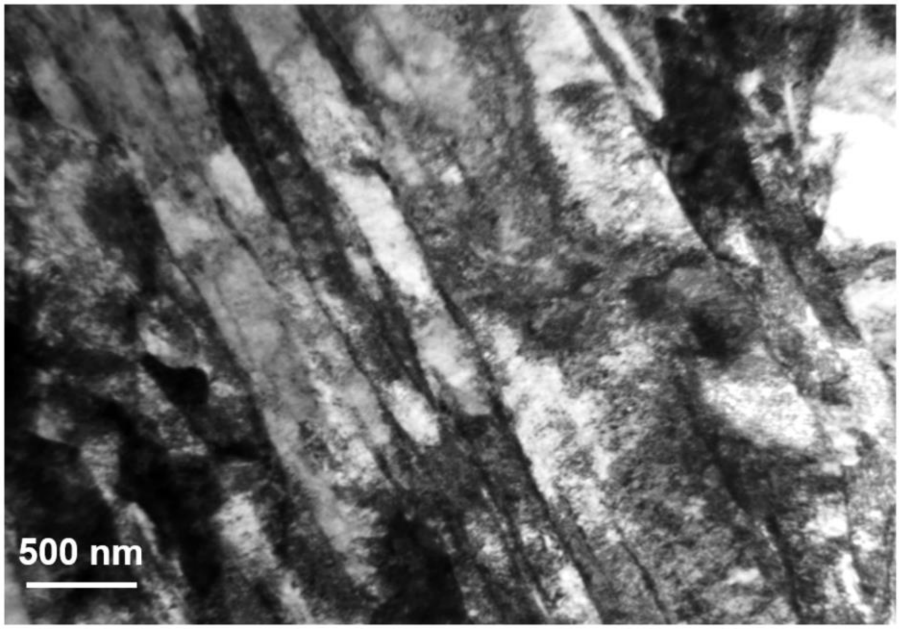

Supplementary Fig. 1 │ Microstructure of the steel. Bright-field TEM image clearly shows the martensite laths in the steel matrix.

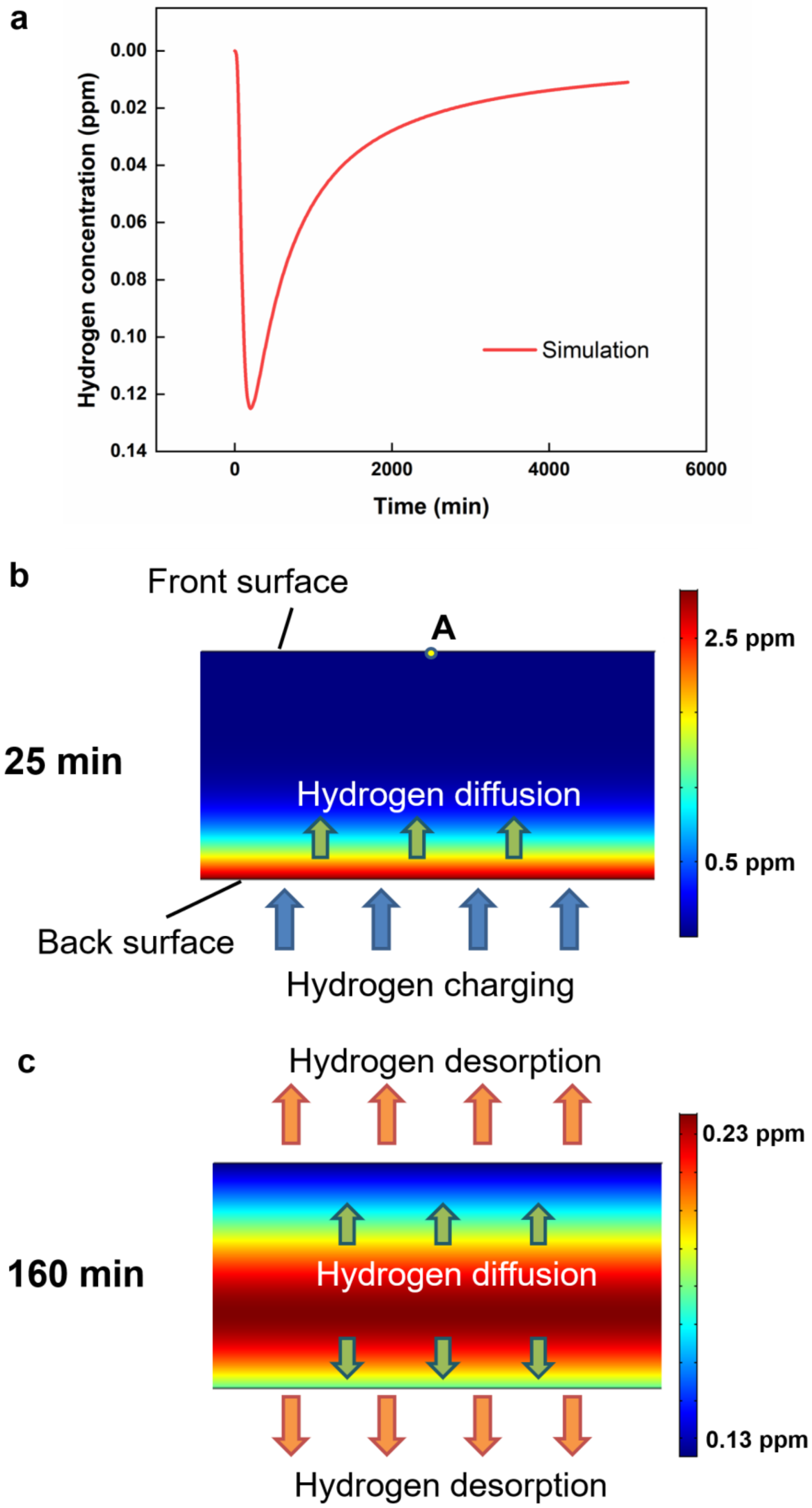
a
Hydrogen concentration (ppm)
0.00
0.02
0.04
0.06
0.08
0.10
0.12
0.14
0
2000
4000
6000
Time (min)
Simulation
b
Front surface
A
25 min
Hydrogen diffusion
Back surface
Hydrogen charging
2.5 ppm
0.5 ppm
c
Hydrogen desorption
160 min
Hydrogen diffusion
Hydrogen desorption
0.23 ppm
0.13 ppm

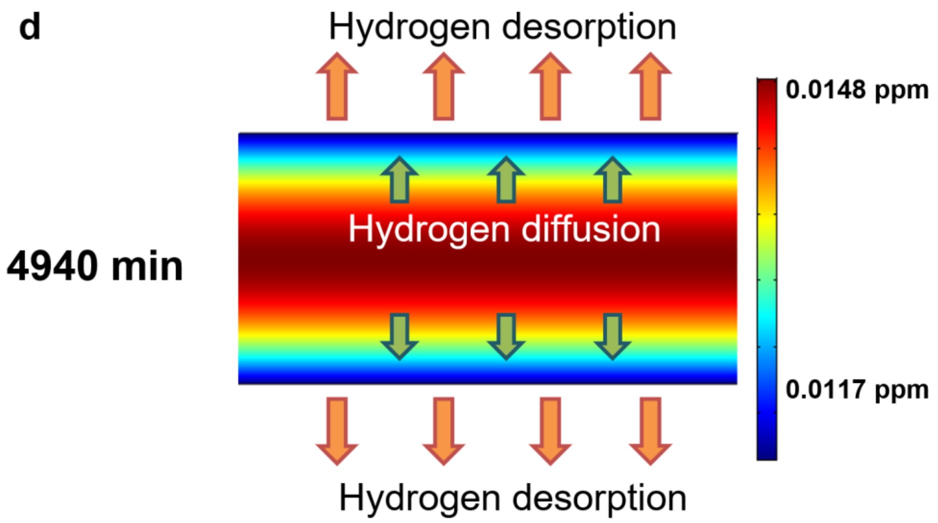

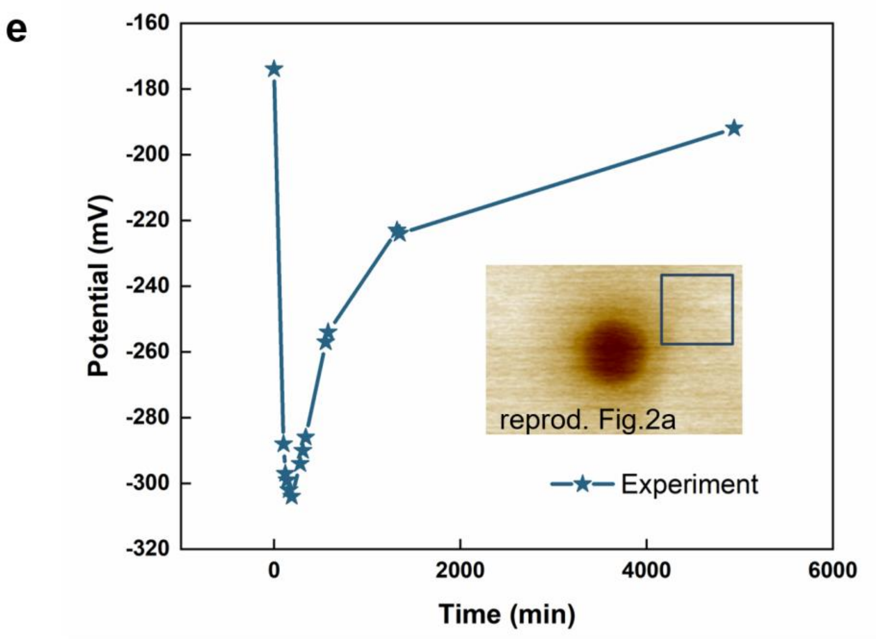

Supplementary Fig. 2 │ The simulated hydrogen concentration evolution within the sample and measured Volta potential difference (simply referred to as "potential" in the following) evolution on the matrix. a. Simulated hydrogen concentration on the sample front surface based on the Fick's second law. **b-d.** Sequential images showing the simulated hydrogen distributions in the sample cross-section. Point A in panel (**b**) is where the simulation data in panel (**a**) is collected. **e.** The measured potential evolution of the matrix (boxed area in the inset). Details of the simulation method is described in Supplementary Method 1.

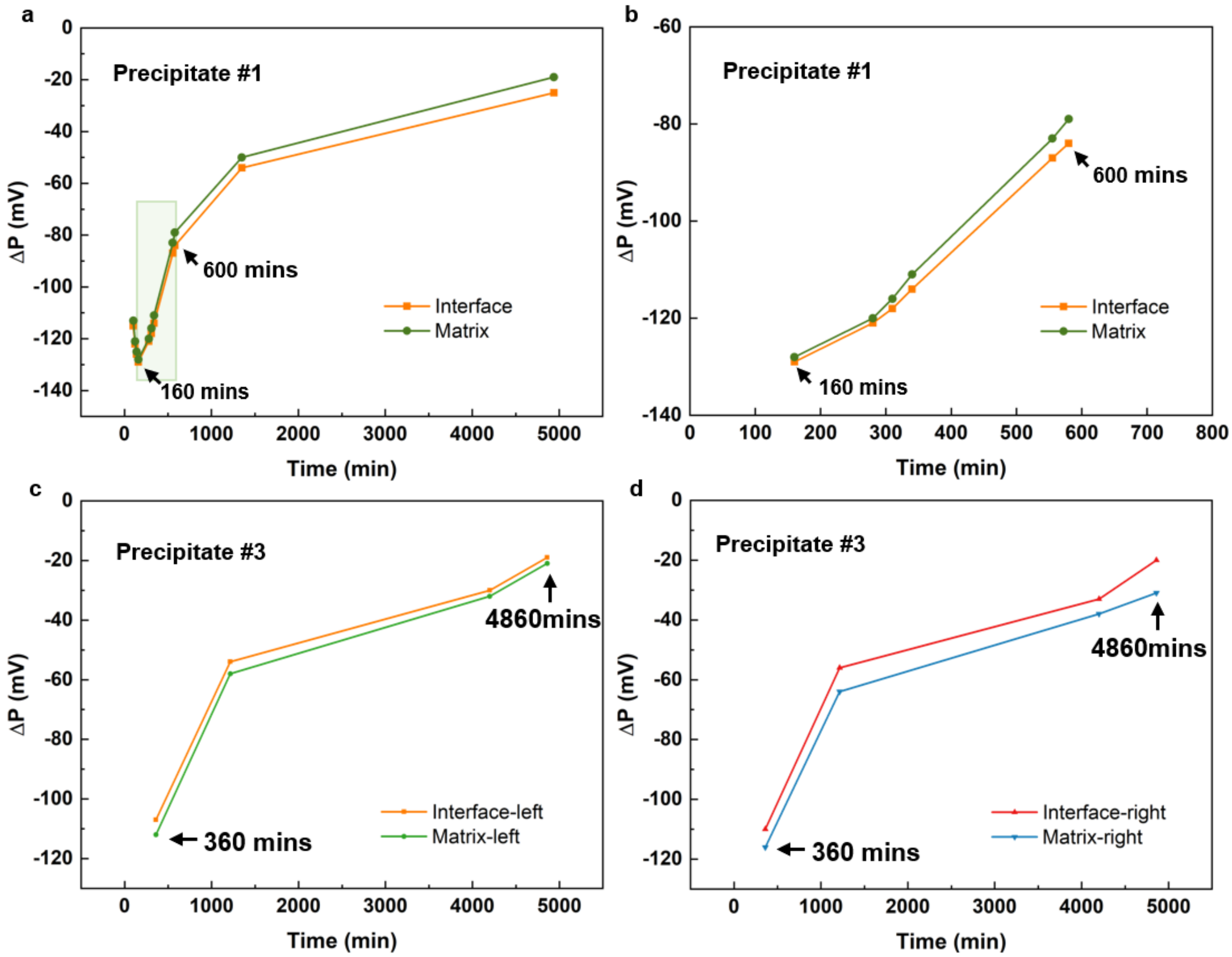

Supplementary Fig. 3 | The temporal evolution of the measured potential drop (ΔP) on the interface and matrix. ΔP is defined by ΔP = P(*t*) - P(0), wherein P(*t*) and P(0) are measured potential at *t* min after H charging and uncharged state. As shown in panel (**a**), both potential of the matrix and potential nearby the border of precipitate #1 rapidly decreased in roughly the same speed before 160 minutes, implying that H permeation rate was likely the same at both sites in the time interval. This is reasonable given the limited trapping sites in the border vicinity which can be quickly filled during the H permeation. When surface H concentration significantly dropped much later, the trapping sites gradually released H into the oxide film, rendering a slightly lower potential at the interface than at the matrix afar and gradually developed since 160mins as shown in panel (**b**) which is enlarged from the boxed region in panel (**a**). Whereas, as for precipitate #3, potential of the border vicinity recovered quickly and always showed higher potential than that of the matrix from 360mins to 4860mins (**c**, **d**), demonstrating that the area nearby the border of precipitate #3 does not trap more H than the matrix.

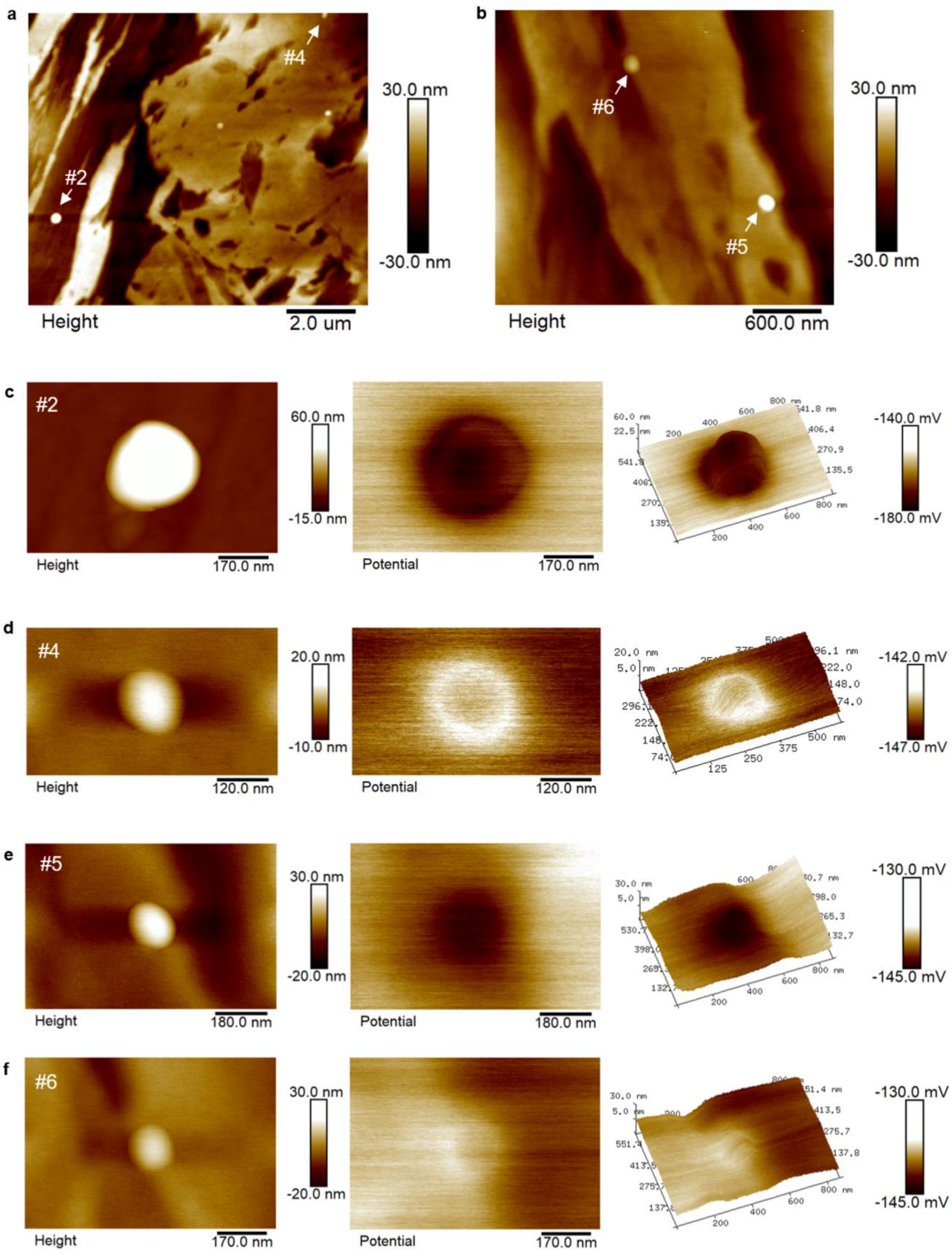
a
#4
#2
30.0 nm
-30.0 nm
Height
2.0 um
b
#6
#5
30.0 nm
-30.0 nm
Height
600.0 nm
c
#2
60.0 nm
-15.0 nm
Height
170.0 nm
Potential
170.0 nm
-140.0 mV
-180.0 mV
d
#4
20.0 nm
-10.0 nm
Height
120.0 nm
Potential
120.0 nm
-142.0 mV
-147.0 mV
e
#5
30.0 nm
-20.0 nm
Height
180.0 nm
Potential
180.0 nm
-130.0 mV
-145.0 mV
f
#6
30.0 nm
-20.0 nm
Height
170.0 nm
Potential
170.0 nm
-130.0 mV
-145.0 mV

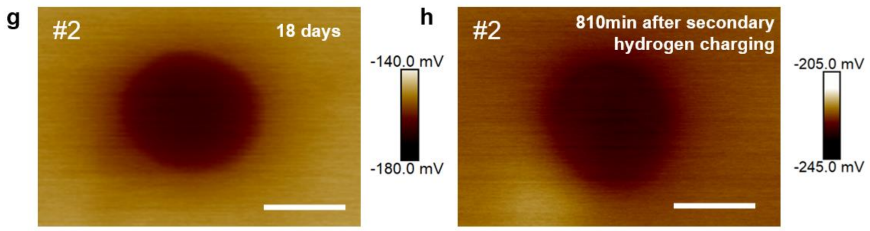

Supplementary Fig. 4 │ In situ scanning Kelvin probe force microscopy (SKPFM) results of more precipitates. **a-b.** AFM topography map of the nanosized precipitates inside the martensite matrix. **c-f.** Topography maps and corresponding potential images of 4 additional precipitates after hydrogen charging. **g-h.** Potential images of precipitate #2 on the 18th day after the H pre-charging and 810mins after re-charging H. Scale bars in (**g**, **h**), 200nm.

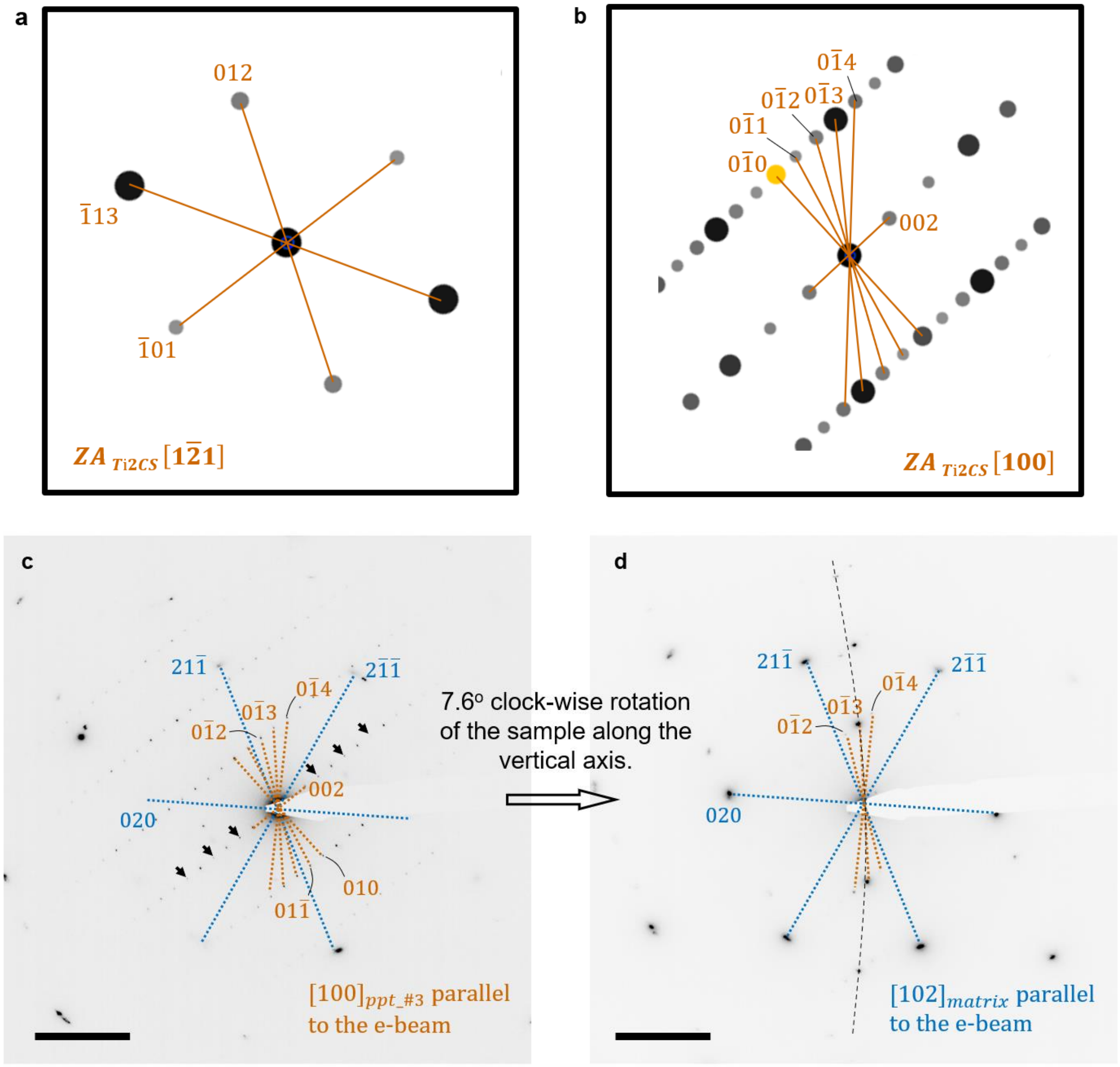

Supplementary Fig. 5 │ Simulated diffraction patterns and mutual orientation analysis of the precipitates. a-b. Simulated diffraction patterns of the $[1\bar{2}1]$ axis and [100] axis based on the Ti_2CS structure. The diffraction pattern simulation was performed by using a JEMS software. **c-d.** Mutual orientation analysis between precipitate #3 and the surrounding martensite matrix. Arrows in panel (c) indicate forbidden reflections visible due to double diffraction. Scale bars in (**c&d**), 5 nm^{-1}.

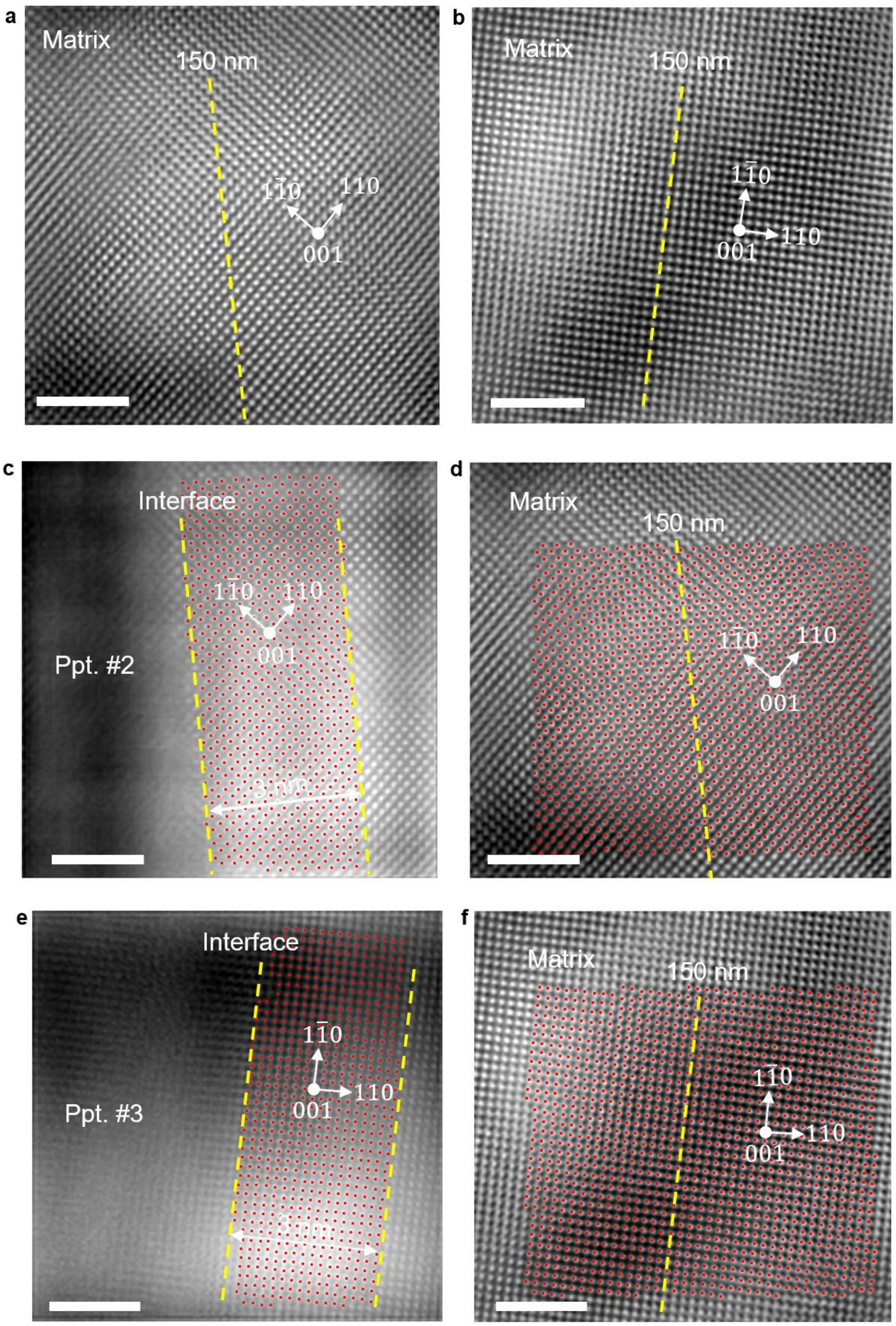

Supplementary Fig. 6 ｜Filtered atomic-resolution high-angle annular dark field scanning transmission electron microscopy (HAADF-STEM) images of the matrix nearby the interface and afar. a-b. The filtered atomic-resolution HAADF-STEM images of the martensite

matrix of ~150nm away from the interface of precipitate #1 and precipitate #3. **c-f.** Images that are reproduced from Figs. 6a&b and panels **a**&**b** here with superposed markers of atomic column positions (red dots) within the area of interest as determined by the CalAtom software. All scale bars, 2nm.

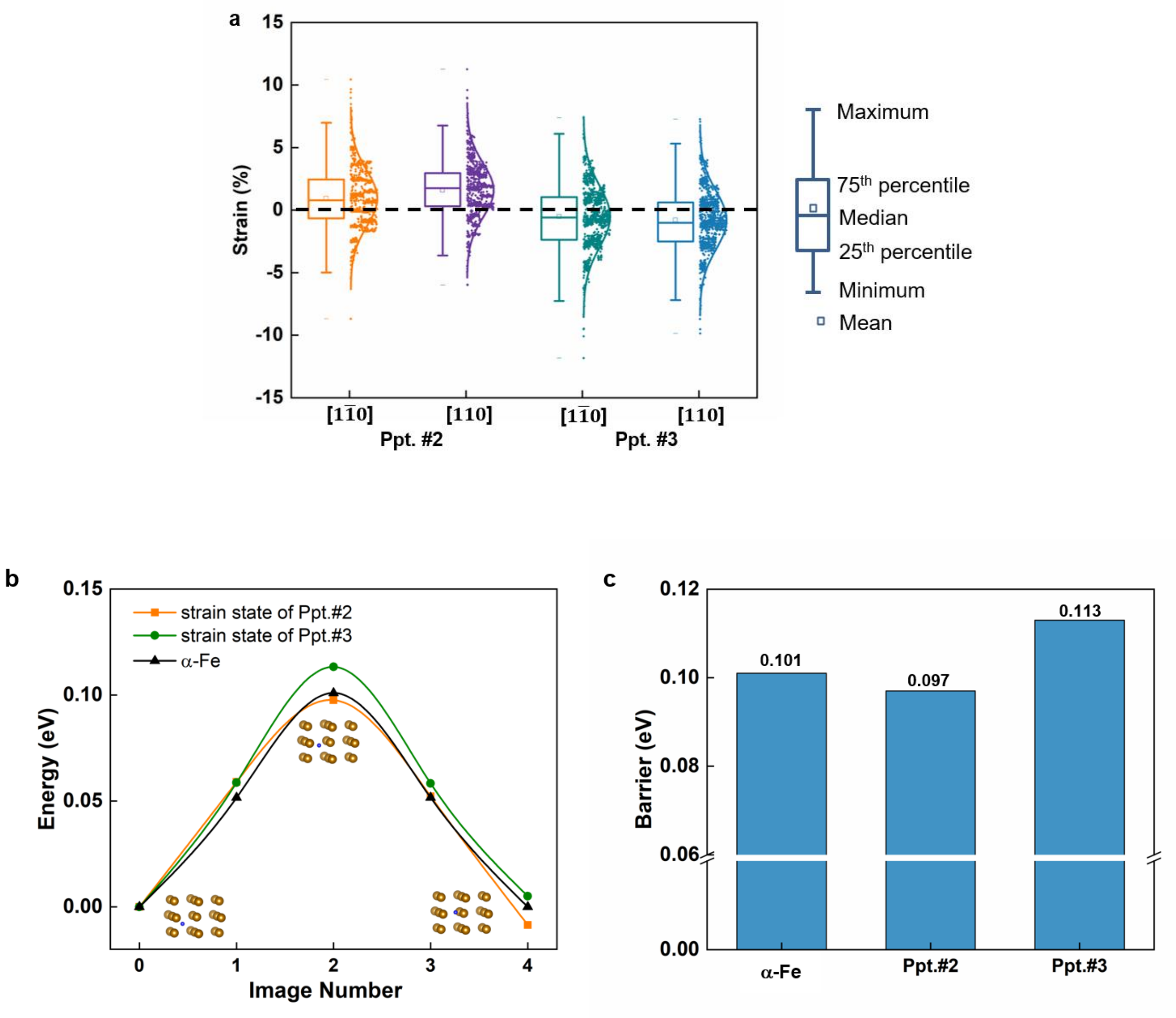

Supplementary Fig. 7 │ Statistical analysis of the strains and energy barrier of hydrogen diffusion in unstrained and strained α-Fe lattices. a. Box-charts of the measured strains of precipitate (Ppt.) #2 and precipitate (Ppt.) #3. **b-c.** Calculated diffusion barriers of H atom in α-Fe lattice with different strain states.

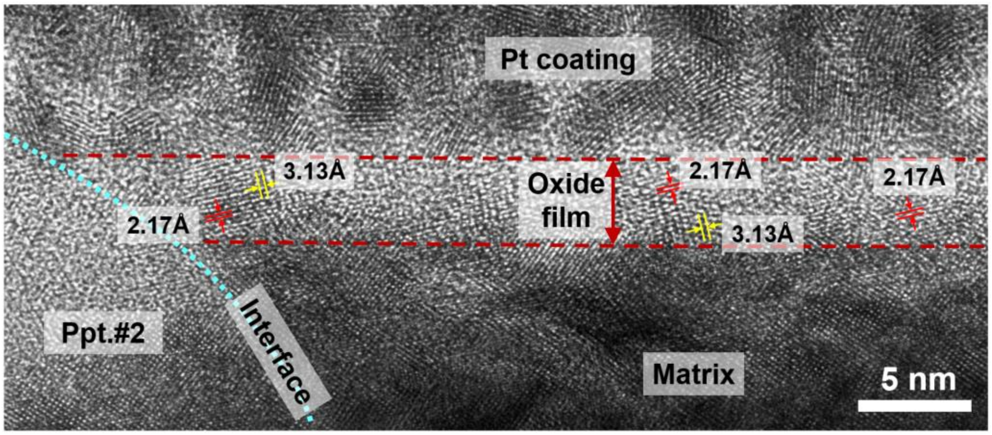

Supplementary Fig. 8 | High-resolution transmission electron microscopy (HRTEM) image of the surface oxide film. The cyan dot line indicates the interface between the precipitate #2 and the matrix. The red dash lines indicate the boundaries of the oxide film.

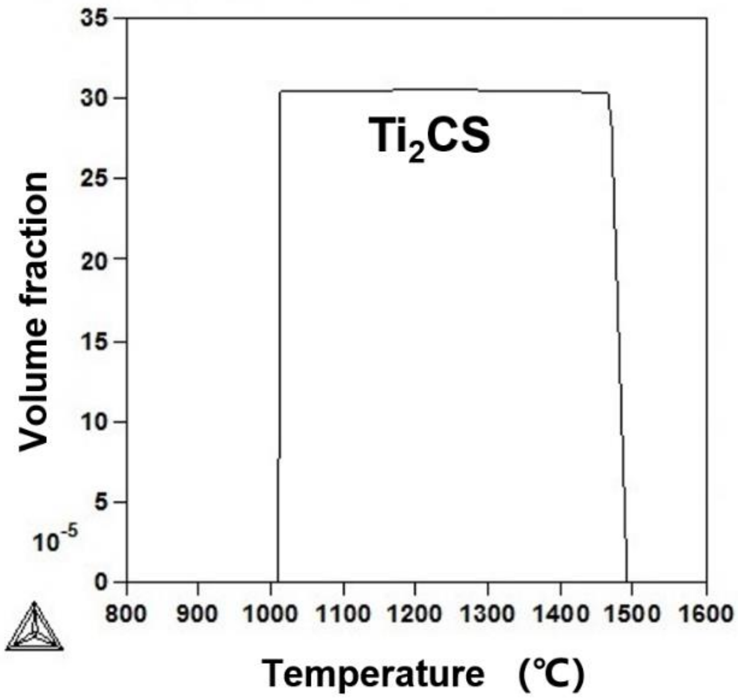

Supplementary Fig. 9 │ Formation temperature of the Ti_2CS precipitates. The volume fraction—temperature plot of the Ti_2CS precipitate is calculated by using a Thermo-Calc software.

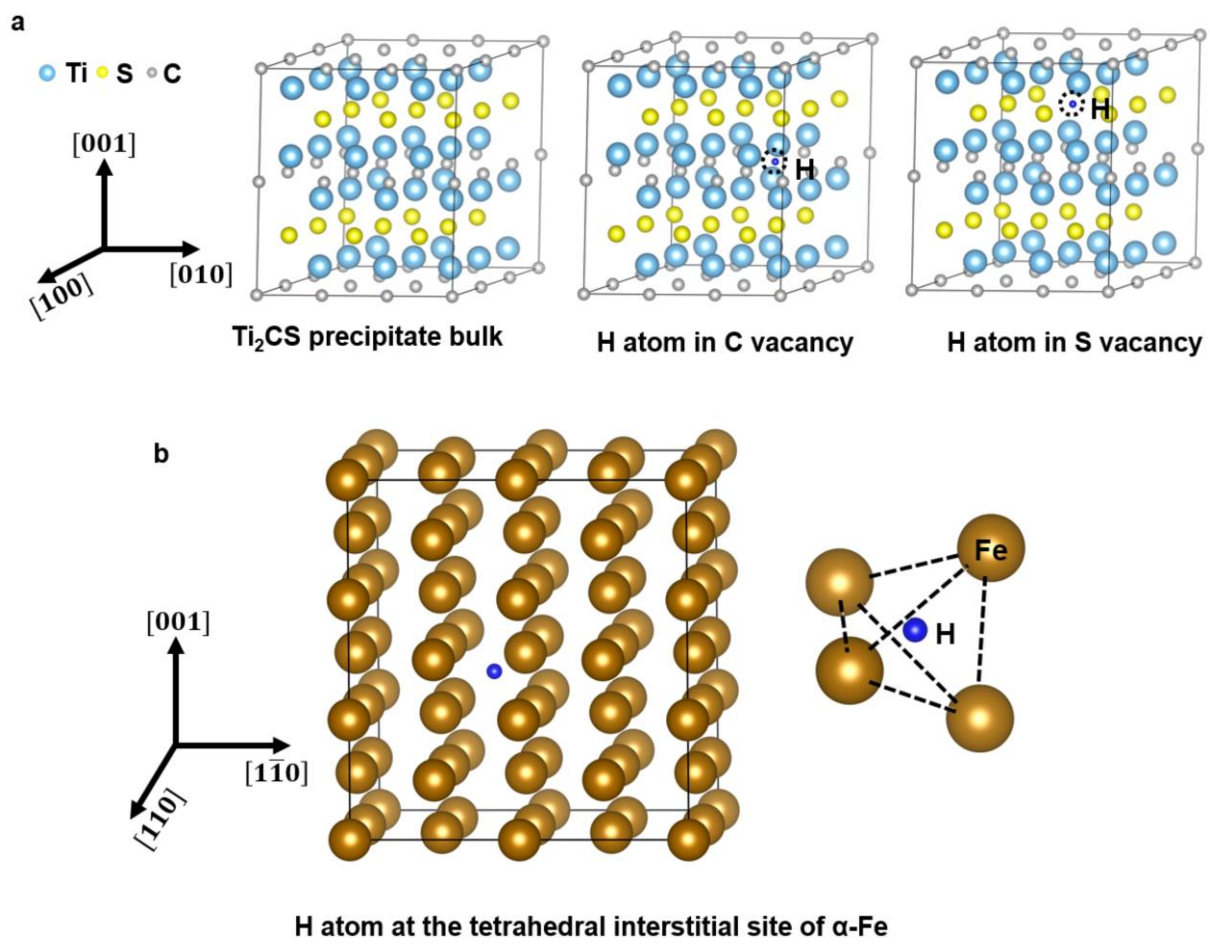

Supplementary Fig. 10 │ Atomic structures used in the density function theory (DFT) calculations on hydrogen solvation energy at the various trapping sites. a. The atomic models of the Ti_2CS precipitate without and with H atom in the C and S vacancies for the DFT calculations. **b.** The atomic model of α-Fe with a H atom in the tetrahedral site for the DFT calculation.

Supplementary Table 1 | Elemental composition of the steel (in wt.%)

C	Si	Mn	P	S	Cr	Ni	Cu	Mo	Ti	V	Nb	Al	Fe
0.077	0.29	0.72	0.010	0.007	2.34	0.80	1.85	1.03	0.10	0.27	0.008	0.055	Bal.

Supplementary Method 1

A COMSOL software was used to simulate the hydrogen diffusion in the plate-shaped sample (with a 490×1000um rectangle shape cross-section) by using the physical mode of "Transport of Diluted Species". Following the experimental procedure, we simulated the following two processes: (1) electrochemical charging of hydrogen at a constant current density for 25mins on the sample back surface; (2) the subsequent spontaneous diffusion of hydrogen within the sample.

In the first process, the hydrogen concentration on sample back surface is set as a constant (C_0), under a constant current density (i_{ss}) in the electrochemical charging; and the two parameters can be related by [1],

$$C_0 = \frac{L i_{ss}}{D F \rho} \tag{1}$$

where L is the sample plate thickness, F is the Faraday constant, ρ is the density of the sample (~7.88 g cm^{-3} for steel) and D is the hydrogen diffusion coefficient ($\sim 3.3 \times 10^{-8}$ cm^2 s^{-1} for our sample). Therefore, the C_0 is calculated to be 2.96 ppm.

By the end of the hydrogen charging process, the hydrogen distribution in the sample can be estimated by [2]:

$$C(x, \mathrm{t}_0) = \mathrm{C}_0\left(1 - erf\left(\frac{x}{\sqrt{4\mathrm{Dt}_0}}\right)\right) \tag{2}$$

where x is the distance along the sample thickness direction ($0 \leq x \leq d$), t_0 is the charging time (i.e. 25 mins).

In the second process, the pre-charged hydrogen will spontaneous diffuse within the sample, driven by concentration gradients. The hydrogen concentration (C') can be described by:

$$\frac{\partial C'}{\partial t} = D\frac{\partial^2 C'}{\partial x^2} \tag{3}$$

The initial condition is defined as the final state of the first process, i.e., $C'(x, \mathrm{t}_0) = C(x, \mathrm{t}_0)$.

$$C'(x, t) = \mathrm{C}_0\left(1 - erf\left(\frac{x}{\sqrt{4\mathrm{D}t}}\right)\right) \tag{4}$$

The desorption rate of hydrogen atoms is set to be proportional to the square of hydrogen concentration [3], i.e.

$$D \frac{\partial C\prime}{\partial x} |_{x=0,\mathrm{d}} = 2kC'^2 \quad (5)$$

where k is the recombination rate of hydrogen atoms which is set as 5×10^{-9}. Note that, in reality, hydrogen atoms desorb by both recombination and oxidation on the sample surface, and the recombination rate of hydrogen atoms is very sensitive to the surface condition and likely changes as the surface oxide film changes with H infusion. Therefore, the above formulation can only give a qualitative estimation on the trend of hydrogen concentration evolution.

By setting $x = \mathrm{d}$, temporal evolution of hydrogen concentration at the sample front surface is obtained (Supplementary Fig. 2a), which incidentally shows the same trend as the measured potential evolution (Supplementary Fig. 2e), albeit quantitative correlation between the hydrogen concentration and the potential is yet to be explored.

Supplementary Note 1

The precipitates are dispersed in a large surface area; and therefore, it takes time to switch the SKPFM scan from one local region to another. Therefore, at a certain moment, we can only work on one potential mapping of a single precipitate. Actually, we focused on precipitate #1 and #3 in our in-situ SKPFM experiment, and recorded their potential evolution after hydrogen charging (see the results in Fig. 2). We only probed the precipitate #2 at 240hrs after the hydrogen charging which showed a hydrogen-trapping characteristic similar to that of precipitate #1 (Supplementary Fig. 4c and Fig. 2d). Unfortunately, precipitate #1 was destroyed in the subsequent FIB process, since the precipitate was embedded in the Pt protection layer and had a risk of being sectioned during the FIB thinning process. Therefore, we chose precipitate #2 which showed a similar hydrogen-trapping characteristic as precipitate #1 for the TEM analysis, while used the in situ SKPFM result of precipitate #1 in Figure 2 of the manuscript.

Supplementary Discussion 1

Per the SKPFM results in Figure 2, both precipitates interiors show lower potentials than the martensite matrix before H charging. As H reaches the sample surface, the potential decrease of the precipitate #1 and the precipitate #3 are similar (Figs. 2d&i). In fact, we cannot exclude the possibility that H flux may enter the Ti_2CS precipitates and lead to the observed potential drop at the precipitate surface. Although it is really hard for H to dissolve in defect-free precipitates, H atoms may diffuse through the precipitates presumably under the combined actions of hydrogen concentration, defects and strain fields.

Previous experimental results on whether electrochemically-charged H can be trapped within the incoherent carbide precipitates are controversial. For instance, atom probe tomography results solidly reveal deuterium segregation in incoherent VMoNbC precipitates [4]. While, TDS data indicates trivial H trapping by the incoherent TiC precipitates [5]; in this study, no solid evidence was provided to exclude the possibility of H being able to permeant through the interface and the incoherent precipitates.

In the kinetic process of H entering the precipitates, H atoms have to jump from the trapping mechanisms on the interface into the precipitate interior. Previous density function theory calculations indicate that coherent and semi-coherent interfaces of carbides (TiC, VC, NbC) with the matrix can trap H, rendering high kinetic energy barrier for H diffusion into the carbides [6,7]. These findings do not necessarily apply to the incoherent interfaces between Ti_2CS and α-Fe in our case. In principle, the local structures of the incoherent interfaces are intrinsically more diverse than the coherent and semi-coherent interfaces; in addition to sites with spacious vacancies that can trap H atoms, there should also be interfacial sites that do not trap H or even exclude H atoms. At these sites, the kinetic energy barrier for H atoms to enter the precipitates is probably much lower. In other words, local regions on the incoherent interface may allow easier permeation of H into the precipitates. Moreover, the role of defects within the precipitates cannot be disregarded which could facilitate the process of hydrogen diffusion through the precipitates.

Above all, H could have entered the incoherent Ti_2CS precipitates and led to the potential drop on the surface of the precipitates.

Moreover, it might be carbide or a mix of carbide and oxide on the precipitates surface. Due to the most likely different surface films on the matrix and on the precipitate, it is not rigorous to compare their hydrogen trapping ability by using the potential. Ageing of the film on top of the precipitate is likely trivial in the short duration of each SKPFM scan and will lead to potential increase which cannot explain the apparent potential drop between consecutive SKPFM scans.

In principle, H atoms entering the precipitates will not affect the capture and diffusion of H on the interfaces since they have to go through and interact with the interfaces prior to entering the precipitates. If the interface does trap H, the interfacial traps will be filled as H atoms diffuse into the precipitate. As for the interface that excludes H, H atoms may diffuse through the interface without being trapped by the interface.

Additionally, since the solution energy of H in the precipitate is higher than that in the matrix, other than desorption from the precipitate surface by either recombination into H_2 or react with O_2 in air, a small amount of H atoms inside the precipitates can flow back into the interface as H desorbs from the sample. As such, H presence inside the precipitates may affect the potential evolution on the interface. However, based on the following discussion, we believe that the backflow of H from the precipitates into the interface does not alter our interpretations.

(1) Based on our DFT calculations, the lowest solution energy of H atoms within the Ti_2CS lattice is ~1.04eV which is much higher than that in α-Fe (~0.23eV). As such, the total amount of H that could enter the precipitate is limited; additionally, many of the H atoms that do enter the precipitates may desorb through recombination into H_2 or reaction with O_2 in air on the surface. In other words, the amount of H atoms that flow from the precipitate into the interface should be very limited.

(2) The measured potential evolution on the interface of precipitate #1 and matrix (Figs. 2a-d) can only be interpreted by the H-trapping interface. The backflow of H from the precipitate supplies more H atoms into the interfacial traps which prompts the formation of relatively low potential (dark ring) on the interface. If the interface does not trap H (or exclude H), one would expect an even (or higher) potential on the interface compared to the matrix during the H ingression at 160mins.

(3) The backflow of H from the precipitate supplies more H atoms into the interface which should prompt the H activity on the H-trapping interface and enhance the “dark ring” feature in the SKPFM image. However, as has been demonstrated in the above reply, the potential on the interface of precipitate #3 and matrix (Figs. 2f-i) was always higher than that of the matrix, indicating that the interface did not trap H.

Supplementary Discussion 2

Due to the following reasons, the oxide films in the vicinity of the interface and on the matrix should qualitatively be the same, and respond to H uptake in the same manner.

1) The steel sample is low-alloyed and hence the oxide film should mainly be composed of iron oxides, hydroxides, and oxyhydroxides.

2) The EDS data shows trivial chemical segregation at the interface (Figs. 3h&i) and hence the matrix nearby the interface and far away from the interface should have the same composition and surface oxides.

3) Referring to the HRTEM images of the surface oxide film near the border and relatively far away from the border, the film thickness and lattice spacings of the crystalline species within the oxide film are generally the same (Supplementary Fig. 8).

In addition, defects within the oxide film (such as vacancies and voids) may also affect the potential response to H [8]. In our study, since all potential responses are captured on the same sample and the oxide films are formed under the same condition (refer to the sample preparation process in the above response), the types and content of defects in oxide films are reasonably the same. Moreover, the potential differences between the interface and the matrix are clearly much larger than the potential fluctuations (Figs. 2e&j), demonstrating that the observed feature cannot be ascribed to the variation of defects in the oxide film.

In fact, the oxide film can change during the SKPFM experiment, due to following reasons. (1) At 26℃ and 38%r.h. humidity, thickness of the oxide film increases very slowly due to continuous oxidation. (2) H can be irreversibly trapped by defects (such as vacancies and boundaries) within the oxide film, which cannot be completely released at room temperature. However, as shown in Supplementary Fig. 4h, when the sample was charged with H for the second time, the potential response as detected on the sample surface is similar to what has been observed in the prior test; this implies that the above-mentioned changes do not significantly alter the response of oxide film to H activity.

The initial slightly higher potential at the border of precipitate #3 (Fig. 2f) may be attributed to the preexisting H in the oxide film which may have been introduced during the mechanical polishing process wherein water was used as a coolant. As such, the sample was not literarily H-free before the H-charging step; a small amount of H may have led to the “bright ring” feature in the initial SKPFM scan. Similarly, a faintly low potential can be seen on the border of precipitate #1 in the initial SKPFM scan (Fig. 2a).

Supplementary Discussion 3

If the interface of the precipitate #3 as shown in Figs. 2f-i traps hydrogen, as the diffusible and shallowly-trapped hydrogen gradually effuse from the matrix, the potential at the border area would eventually become lower than the matrix afar due to the slow release of hydrogen from the trapping site. On the contrary, the relatively high potential (“bright” ring feature) at the interface persists for more than 10 days. Additionally, the opposite interpretation could not fairly explain what was observed in Figs. 2a-d. Specifically, if the interface of precipitate #1 does not trap hydrogen, the potential at the border region should by no means be lower than the matrix afar when hydrogen within the sample has substantially effused; while, our observation clearly shows the contrary (see the obvious “dark” ring feature in Fig. 2d).

In principle, as hydrogen can be captured by the traps, there should be a stage when the region right above the trapping site show a relatively higher potential than the matrix. However, this stage should be transitional and quickly disappear as soon as the traps are filled up. Given that the number of trapping sites on the border of a single precipitate particle (e.g., precipitate #1) are limited, they could be filled up very quickly as the hydrogen arrives. In other words, the time window of the transitional stage should be narrow; this is likely why we did not capture the transitional stage in our SKPFM experiment (Figs. 2a-d). Based on the observation, such transitional stage has already ceased by 100mins (when the first SKPFM image was acquired in Supplementary Fig. 3a). Moreover, referring to the energy barrier of hydrogen diffusion in the strained α-Fe lattice (Table 1 and Supplementary Figs. 7b-c), the tensile strains with lower the H solution energy renders a lower energy barrier for H diffusion than the compressive stains. In other words, hydrogen “traps” do not necessarily impede H diffusion therein.

Indeed, given sufficiently strong trapping of H by the interface, the release of H at the very local will be trivial when most H has escaped from the sample, rendering no observable potential drop at the border of the precipitate. However, based on our TEM characterization, a type of feature that affect H distribution is the elastic strain nearby the border region, which is not strong H traps [9]. Consistently, the border of precipitate #1 shows a lower potential than the matrix at 4940mins, as H atoms within the tensile strain field are gradually released into the oxide film overtime.

In addition, what we saw on the 10th day (Supplementary Fig. 4c) was not a “frozen-in” state. As shown in the potential mappings in Supplementary Figs. 4c&g, the potential at the precipitate/matrix interface was still continuously evolving from the 10th day to the 18th day. The “dark ring” feature in the 10th-day scan clearly fades away in the 18th-day scan, which is consistent with the inference that H at the interface has escaped.

Supplementary References

1 Ma, Z., Xiong, X., Chen, L. & Su, Y. Quantitative calibration of the relationship between Volta potential measured by scanning Kelvin probe force microscope (SKPFM) and hydrogen concentration. *Electrochim. Acta* **366**, 137422 (2021).

2 Crank, J. *The Mathematics of Diffusion* (Oxford Univ. Press, Oxford, 1975).

3 Nagumo, M. *Fundamentals of Hydrogen Embrittlement* (Springer, Singapore, 2016).

4 Chen, Y. S. *et al.* Direct observation of individual hydrogen atoms at trapping sites in a ferritic steel. *Science* **355**, 1196-1199 (2017).

5 Wei, F. G. & Tsuzaki, K. Hydrogen absorption of incoherent TiC particles in iron from environment at high temperatures. *Metall. Mater. Trans. A* **35**, 3155-3163 (2004).

6 Shi, R. *et al.* Atomic-scale investigation of deep hydrogen trapping in NbC/α-Fe semi-coherent interfaces. *Acta Mater.* **200**, 686-698 (2020).

7 Ma, Y. *et al.* A first-principles study on the hydrogen trap characteristics of coherent nano-precipitates in α-Fe. *Int. J. Hydrog. Energy* **45**, 27941-27949 (2020).

8 Evers, S., Senoz, C. & Rohwerder, M. Hydrogen detection in metals: a review and introduction of a Kelvin probe approach. *Sci. Technol. Adv. Mater.* **14**, 014201 (2013).

9 Wei, F. G. & Tsuzaki, K. Quantitative analysis on hydrogen trapping of TiC particles in steel. *Metall. Mater. Trans. A* **37**, 331-353 (2006).
